# LILRB1-directed CAR-T cells for the treatment of hematological malignancies

**DOI:** 10.1038/s41375-025-02580-z

**Published:** 2025-04-05

**Authors:** Katsiaryna Marhelava, Klaudyna Fidyt, Monika Pepek, Marta Krawczyk, Christopher Forcados, Agata Malinowska, Bianka Swiderska, Narcis Fernandez-Fuentes, Natalia Czerwik, Iwona Baranowska, Agnieszka Krzywdzinska, Lukasz Sedek, Lukasz Slota, Bartosz Perkowski, Alicia Villatoro, Thibault Leray, Ewa Lech-Maranda, Pablo Menendez, Else Marit Inderberg, Sébastien Wälchli, Magdalena Winiarska, Malgorzata Firczuk

**Affiliations:** 1 Department of Immunology, Mossakowski Medical Research Institute, Polish Academy of Sciences, Warsaw, Poland; 2 Department of Immunology, Medical University of Warsaw, Warsaw, Poland; 3 Josep Carreras Leukemia Research Institute, Barcelona, Spain; 4 Doctoral School of Translational Medicine, Mossakowski Medical Research Institute, Polish Academy of Sciences, Centre of Postgraduate Medical Education, Warsaw, Poland; 5 Translational Research Unit, Section of Cellular Therapy, Department of Oncology, Oslo University Hospital, Oslo, Norway; 6 Institute of Clinical Medicine, Faculty of Medicine, University of Oslo, Oslo, Norway; 7 Mass Spectrometry Laboratory, Institute of Biochemistry and Biophysics, Polish Academy of Sciences, Warsaw, Poland; 8 Laboratory of Immunophenotyping, Institute of Hematology and Transfusion Medicine, Warsaw, Poland; 9 Department of Microbiology and Immunology, Medical University of Silesia in Katowice, Zabrze, Poland; 10 Department of Pediatric Hematology and Oncology, Medical University of Silesia in Katowice, Zabrze, Poland; 11 Department of Hematology, Institute of Hematology and Transfusion Medicine, Warsaw, Poland; 12 Centro de Investigación Biomédica en Red-Oncología, Instituto de Salud Carlos III, Madrid, Spain; 13 Red Española de Terapias Avanzadas (TERAV), Instituto de Salud Carlos III, Madrid, Spain; 14 Institució Catalana de Recerca i Estudis Avançats (ICREA), Barcelona, Spain; 15 Department of Biomedicine, School of Medicine, University of Barcelona, Barcelona, Spain; 16 Institut de Recerca Hospital Sant Joan de Déu–Pediatric Cancer Center Barcelona (SJD-PCCB), Barcelona, Spain

**Keywords:** Immunotherapy, Preclinical research, Cancer immunotherapy

## Abstract

CD19 CAR-T cells have established a new standard for relapsed/refractory B-cell malignancies. However, the treatment fails in 50% of patients, often due to CD19 antigen loss. Alternative immunotherapies targeting other antigens are being tested but show limited efficacy, especially in cases of lineage switching or loss of B-cell phenotype, highlighting the need for novel targets. Herein, we identified leukocyte-immunoglobulin-like-receptor-B1 (LILRB1, CD85j) as a novel target for CAR-T cells through cell surface proteomics on patient-derived samples of high-risk B-cell acute lymphoblastic leukemia (B-ALL). LILRB1, an immune inhibitory receptor, is normally expressed only on monocytes and B-cells. We observed stable LILRB1 expression in B-ALL and B-cell non-Hodgkin lymphoma (B-NHL), even after CD20/CD19-based immunotherapies. LILRB1 CAR-T cells showed antigen-specific antitumor activity in vitro against B-ALL/B-NHL cells, including those resistant to CD19 CAR-T-cells, and in vivo in B-ALL xenografts. Additionally, we identified LILRB1 in monocytic acute myeloid leukemia (AML) and demonstrated LILRB1 CAR-T cell cytotoxicity against AML cell lines in vitro and in vivo. These findings establish LILRB1 as a novel target for cancer immunotherapy and show evidence for the preclinical efficacy of LILRB1 CAR-T cells against haematological malignancies, including cases resistant to previous lines of immunotherapy, thus holding promise for further clinical development.

## Introduction

CD19-targeting chimeric antigen receptor-modified T cells (CD19 CAR-T cells) have emerged as a breakthrough therapy for relapsed/refractory (r/r) B-cell malignancies, establishing a new standard of care. However, CD19 loss, a well-described mechanism occurring in 30–70% of B-cell acute lymphoblastic leukemia (B-ALL) [1–3] and in 20–30% of B-cell non-Hodgkin lymphoma (B-NHL) cases [4–6] following CD19 CAR-T cell therapy, contributes to relapse and treatment failure. Alternative options are being developed for such patients, and several B-cell-specific antigens are already under investigation in preclinical studies as CAR-T targets [7, 8], including CD22 and CD20 emerging as the most advanced ones. Strategies addressing CD19 antigen escape comprise CAR-T cell approaches simultaneously targeting CD19 along with CD20 and CD22. However, they are effective only when both antigens are sufficiently expressed [9, 10]. Accordingly, in cases where CD22 and CD20 expression is low or downregulated/lost together with CD19 [2, 6, 9, 11, 12], these antigens may no longer be viable, underscoring the need for identification of novel therapeutic strategies and targets. Moreover, targeting B-cell-specific antigens is not effective when resistance to CD19 CAR-T therapy is caused by a transition of tumor cells from a lymphoid to a myeloid phenotype [13, 14]. In that case, targeting antigens present on cells of myeloid origin becomes more attractive. Several such targets have been already identified, including CD33, CD123, Lewis (Le)-Y, and FLT3 [15–17], and CAR-T cells directed against these targets have been designed to recognize tumor cells of strictly myeloid origin. These CAR-T therapies demonstrated efficacy in eliminating acute myeloid leukemia (AML) cells in both in vitro and in vivo models, with some undergoing patient testing [18], but none have yet received clinical approval. Most myeloid cell-specific CARs currently under development target pan-myeloid markers, also expressed in hematopoietic stem/progenitor cells (HSPC) [19], leading to severe myelosuppression and significant toxicity [20]. Consequently, the need for suitable myeloid targets remains unmet. Particularly pressing is the need for novel markers that can serve as alternatives following lineage switching and loss of the B-cell phenotype.

Identifying new targets for CAR-T cell therapy presents a multifaceted challenge. Ideal CAR-T target should be homogenously expressed on malignant cells but not on normal tissues to avoid on-target off-tumor toxicity. However, such antigens are limited, and even clinically validated targets such as CD19 or BCMA do not meet these stringent criteria. Also, methodology is a limitation. Recent advances in proteomic methods combining mass spectrometry (MS) and bioinformatics tools led to the identification of some novel targets that were employed for the generation of novel CAR-T cell therapies in B-ALL, AML, multiple myeloma, and even solid tumors [8, 21, 22]. However, in B-ALL and AML studies, the surfaceome characterization was performed on cell lines, limiting the identification of targets that are not accurately represented by these models.

In this study, we developed a strategy for CAR-T target discovery using B-ALL patient-derived xenograft (PDX) cells as a model. Employing cell surface proteomics combined with MS identification of surface proteins in histone-lysine N-methyltransferase 2 A (*KMT2A)*-rearranged B-ALL PDX samples, followed by extensive tissue-specificity analyses, we identified LILRB1 (also known as CD85j) as a potential novel CAR target. Extensive validation of LILRB1 expression revealed its specific occurrence in B-cell-derived malignancies, including B-ALL and B-NHL. LILRB1 was present even in cases where malignant cells had lost CD19 expression post-CD19 CAR-T cell treatment, as well as in monocytic AML. We generated LILRB1-targeting CAR-T cells and confirmed their antitumor efficacy and safety in pre-clinical settings. Thus, LILRB1 represents an attractive target antigen for cell-based immunotherapy to treat different hematological malignancies, including challenging cases previously treated with other lines of treatment.

## Results

### Cell surface proteomics of B-ALL PDX identifies LILRB1 as a putative immunotherapy target

**Fig. 1:**
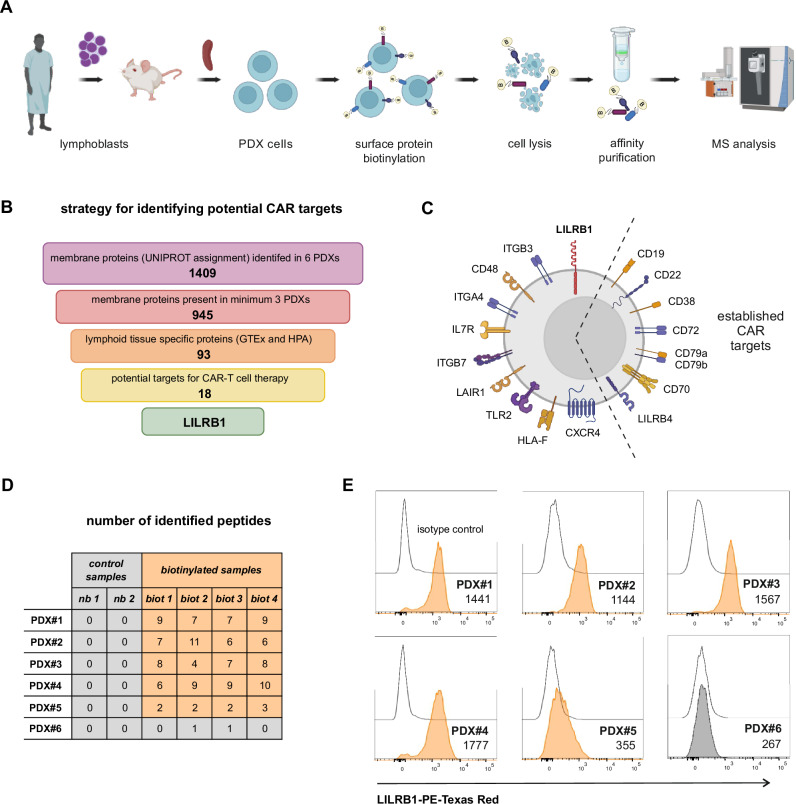
Cell surface proteomics of B-ALL PDX and bioinformatic analyses identify LILRB1 as a candidate target for CAR-T cell immunotherapy. **A** Proteomics workflow for quantifying cell surfaceomes of B-ALL^1^ PDX^2^. **B** A scheme illustrating the selection process of cell surface membrane proteins to identify suitable candidates for CAR^3^-T cell therapy in B-ALL. **C** A graph illustrating 18 surface proteins selected with MS^4^-based method that can serve as targets for CAR-T cell therapy. Already being exploited targets are grouped on the right side of the cell. **D** A table showing the numbers of identified specific peptides derived from the LILRB1 protein across six PDX samples, including all replicates. The data is presented for both non-biotinylated control samples (nb) and biotinylated samples (biot). **E** Flow cytometry histograms showing LILRB1 protein surface levels on the B-ALL PDX samples used for the MS analysis presented in panel D. MFI^5^ for anti-LILRB1 Ab^6^ (clone HP-F1) is shown next to the histograms.

To identify novel targets, we first optimized a method for surfaceome identification using cell surface biotinylation of B-ALL PDX cells, followed by enrichment of biotinylated proteins and quantitative MS (Fig. 1A). This proteomic approach was chosen over transcriptomic methods, as it provides direct evidence of surface protein expression, reducing the likelihood of false positive results from RNA data. We profiled six PDX samples from B-ALL patients with *KMT2A* rearrangements (KMT2A-r PDX) (Supplementary Table 1), a high-risk subtype often showing lineage switch or CD19 loss following CD19-targeted immunotherapy. PDX samples were analyzed in quadruplicate, alongside nonbiotinylated controls. Utilizing label-free quantification in MaxQuant followed by Perseus analysis and filtering for membrane-associated proteins in Uniprot, we identified 1409 membrane proteins (Fig. 1B). Of these, 945 proteins detected in at least three PDX samples were examined for tissue specificity using the Human Protein Atlas and the Genotype-Tissue Expression databases, revealing 93 lymphoid tissue-specific proteins. Following manual curation, we identified 18 potential CAR therapy targets (Fig. 1B, Supplementary Table 2). In particular, we discovered previously unexplored surface proteins as potential CAR targets, such as CD48, IL7R, ITGB7, LAIR1, LILRB1 (Fig. 1C). Notably, this set also included known CAR targets, such as CD19, CD22, CD38, CD70, CD72, CD79A, CD79B, and LILRB4 (Fig. 1C), thus validating our approach. Further literature review of the potential CAR targets excluded proteins expressed in granulocytes and T cells, notably selecting LILRB1, which expression is restricted to monocytes and B cells. LILRB1 was present in 5/6 PDX used for proteomic analysis (Fig. 1D), which was further confirmed by flow cytometry (Fig. 1E). In conclusion, our method identified LILRB1 as a new CAR-T target.

### LILRB1 expression in normal tissues

**Fig. 2:**
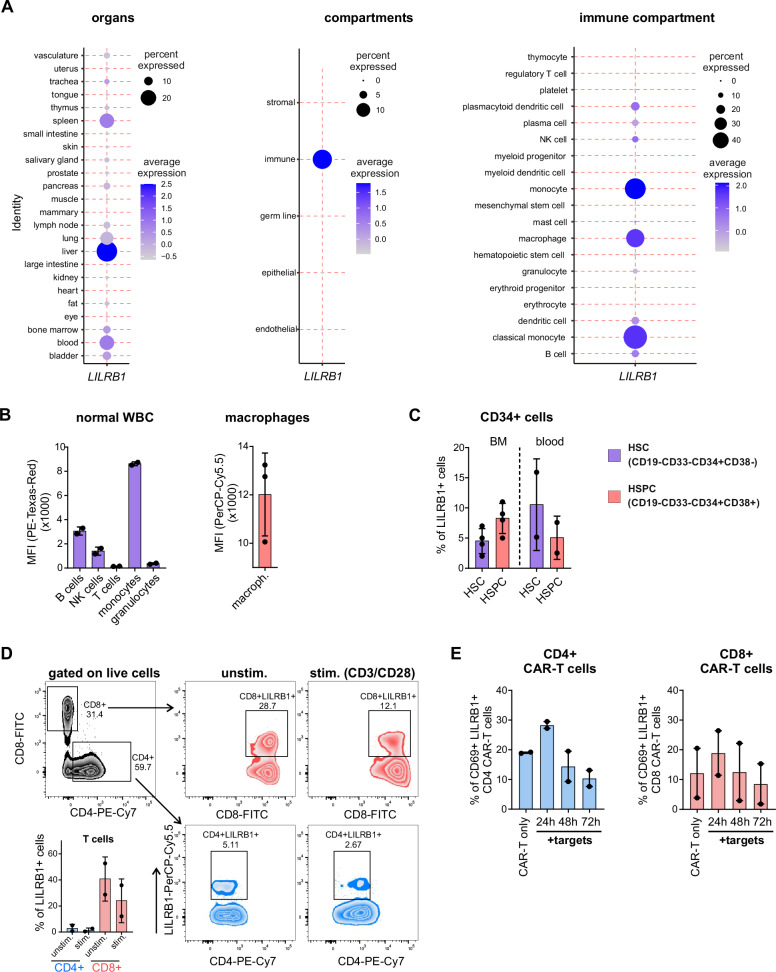
LILRB1 expression is primarily restricted to the lymphoid compartment. **A** *LILRB1* mRNA levels were assessed using the Tabula Sapiens database, containing single-cell transcriptomic data from healthy donors, across different organs (left panel), compartments (middle panel), and immune cell types (right panel). Dot size represents the percentage of cells expressing *LILRB1*, while purple color intensity indicates expression levels. **B** LILRB1 protein levels on various WBC^7^ isolated from two healthy donors were analyzed by flow cytometry using anti-LILRB1 Ab (left panel). LILRB1 surface levels on primary macrophages differentiated from CD14^+^ monocytes of three healthy donors were analyzed by flow cytometry using anti-LILRB1 Ab (right panel). **C** The percentage of LILRB1^+^ cells among defined subpopulations was determined by flow cytometry on normal regenerating BM^8^ samples (*n* = 4) and on apheresis-derived samples collected from individuals who underwent HSC^9^ mobilization with G-CSF^10^ (*n* = 2). Singlets were selected on FSC-H/FSC-A^11^ and mononuclear cells were selected on FSC-A/SSC-A^12^ (not shown). After CD33^+^ and CD19^+^cells exclusion, a population of CD34^+^ cells was gated. Progenitor cells were further divided based on CD38 expression. The level of LILRB1 expression was assessed compared to the appropriate isotypic control (BM), or FMO^13^ control (apheresis blood). **D** Primary human T cells from two healthy donors were stimulated with Dynabeads™ Human T-Activator CD3/CD28 for 2 days. LILRB1 surface levels on unstimulated and stimulated CD4^+^ and CD8^+^ T cells were analyzed by flow cytometry using anti-LILRB1 Ab. **E** CD19 CAR-T cells were co-cultured with Raji target cells for 24, 48, and 72 h. LILRB1 surface levels on CD4^+^ and CD8^+^ CAR-T cells were assessed by flow cytometry using anti-LILRB1 Ab. In (**B**–**E**), clone HP-F1 of the anti-LILRB1 Ab was used.

To validate safety of LILRB1 as a CAR-T target, we first analyzed its expression in normal tissues and across multiple organs using a single-cell human cell atlas from the Tabula Sapiens Consortium [23]. Consistent with previous analyses in the Human Protein Atlas and the Genotype-Tissue Expression databases, *LILRB1* expression was detected by single-cell RNA-sequencing (RNA-seq) in lymphoid tissues such as bone marrow, spleen, blood, and additionally in lungs and liver (Fig. 2A, left panel). However, the detailed analysis revealed *LILRB1* expression exclusively in the immune cell compartment (Fig. 2A, middle panel), primarily in various subpopulations of monocytes, macrophages, and B cells, and the lack of expression in major subpopulations of T cells or hematopoietic stem cells (HSC) (Fig. 2A, right panel, Supplementary Fig. 1). Notably, the *LILRB1* expression in the lungs and liver exclusively stemmed from tissue-resident macrophages and monocytes (Supplementary Table 3). We subsequently confirmed LILRB1 protein presence on healthy donors’ peripheral blood leukocytes, demonstrating the highest expression levels in monocytes and B cells, with much lower expression in NK cells and T cells (Fig. 2B left panel, Supplementary Fig. 2), similarly to already available data [24]. Primary macrophages differentiated in vitro from human peripheral blood monocytes displayed high LILRB1 surface levels (Fig. 2B, right panel). Following transcriptomic data, low LILRB1 protein levels were observed in HSC (CD19^−^CD33^−^CD34^+^CD38^−^) and lineage-restricted progenitors (CD19^−^CD33^−^CD34^+^CD38^+^) from two different sources, namely normal regenerating bone marrow and stem cell apheresis. On average, not more than 10% of HSC and lineage-restricted progenitors expressed LILRB1 (Fig. 2C), suggesting that LILRB1 CAR-T cells and LILRB1-targeted therapy will not ablate normal human hematopoiesis. Given that LILRB1 surface expression in T cells potentially poses a risk of fratricide killing in CAR-T cell manufacturing, we evaluated LILRB1 levels in human T cells activated by CD3/CD28 stimulation. LILRB1 expression was higher in CD8^+^ than in CD4^+^ T cells, decreasing upon stimulation (Fig. 2D). Furthermore, LILRB1 expression remained low and roughly unchanged in CD19 CAR-T cells upon their contact with CD19-positive target cells (Fig. 2E). The pattern of LILRB1 expression was consistent across various CD8^+^ CAR-T cell subsets (naïve, central memory, effector memory, and terminally differentiated T cells, Supplementary Fig. 3). In summary, these analyses indicate that targeting LILRB1 with CAR-T cells is likely to eliminate B-cell and monocyte compartments only and suggest the feasibility of LILRB1 CAR-T manufacturing.

### LILRB1 is expressed in B-cell-derived malignancies

**Fig. 3:**
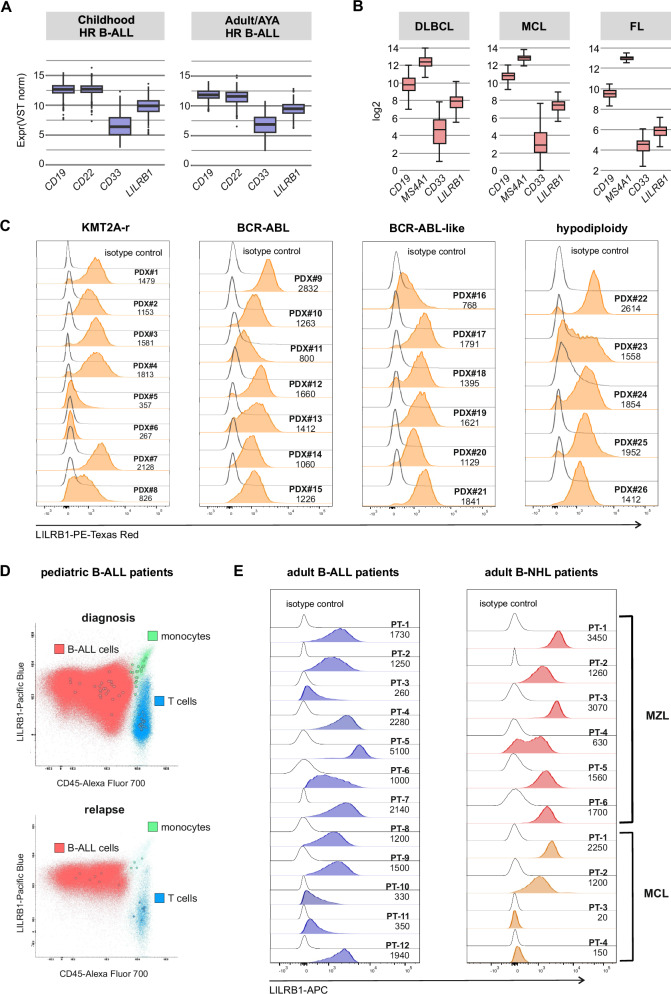
LILRB1 is expressed in malignancies derived from B-cells. **A** Box plot showing mRNA expression levels of *LILRB1*, *CD19*, *CD22*, and *CD33* genes assessed using the St Jude hospital dataset deposited in the EGA^14^ under accession number EGAS00001003266 [59], both in children and adults including young adults with B-ALL. **B** Box plots showing mRNA expression levels of *LILRB1*, *CD19*, *CD22* and *CD33* genes assessed using microarray data downloaded from the GEO^15^ database for: DLBCL^16^ GEO accession number GSE31312 [60]; MCL^17^, GEO accession number GSE93291 [61]; and FL^18^, GEO accession number GSE93261 [62]. **C** LILRB1 expression was evaluated by flow cytometry on B-ALL PDX samples (adult and pediatric) representing various leukemia subtypes (*n* = 26). The cells were stained with anti-LILRB1 Ab. MFI for each sample is shown next to the corresponding histogram. **D** LILRB1 expression was evaluated by flow cytometry in BM samples from B-ALL patients at diagnosis (*n* = 21; upper plot) and relapse (*n* = 7; lower plot) using anti-LILRB1 Ab. Data are presented as a dot plot overlay showing LILRB1 levels in gated B-ALL cells (red) together with monocytes (aquamarine) and T cells (blue) serving as positive and negative reference populations, respectively. Circles in respective colors represent the MFI of B-ALL cells, monocytes, and T cells from individual overlaid cases. **E** LILRB1 expression was evaluated by flow cytometry using anti-LILRB1 Ab on BM samples obtained from adult patients with B-ALL (*n* = 12, left panel) and B-NHL^19^ (*n* = 10, right panel). Histograms show LILRB1 expression levels for gated malignant cells. MFI for each sample is shown next to the corresponding histogram. In panels C-E, clone HP-F1 of the anti-LILRB1 Ab was used.

To explore the utility of LILRB1 for the elimination of tumor cells, we further analyzed its mRNA expression in various pediatric B-cell malignancies from the St. Jude’s B-ALL RNA-seq dataset and lymphoma patients' microarrays data deposited in the Gene Expression Omnibus (GSE31312, GSE93291, GSE93261). In all B-cell-derived malignancies, *LILRB1* expression was lower than that of other B-cell-specific markers such as *CD19* and *CD22* but higher than *CD33* (negative control) (Fig. 3A, B). Subsequently, we evaluated LILRB1 surface protein levels in B-ALL PDX. Notably, LILRB1 was expressed in 24/26 tested PDX samples representing high-risk B-ALL subtypes including KMT2A-r, BCR-ABL1, BCR-ABL1-like and hypodiploid, with typically moderate but uniform expression levels across cell population (Fig. 3C). Additionally, we assessed LILRB1 protein levels in freshly isolated primary samples at diagnosis and relapse in pediatric and adult patients with various B-cell malignancies. In the pediatric cohort, we observed moderate expression in most diagnostic and relapsed B-ALL cells (Fig. 3D, Supplementary Table 4). In adult patients, the prominent expression of LILRB1 was found in most B-ALL and various B-NHL malignant cells (Fig. 3E, Supplementary Table 5). These findings underscore the substantial expression of LILRB1 across various B-cell malignancies, emphasizing its potential as a viable target for immunotherapy.

### LILRB1 is stably expressed in malignant B-cells resistant to previous lines of immunotherapy

**Fig. 4:**
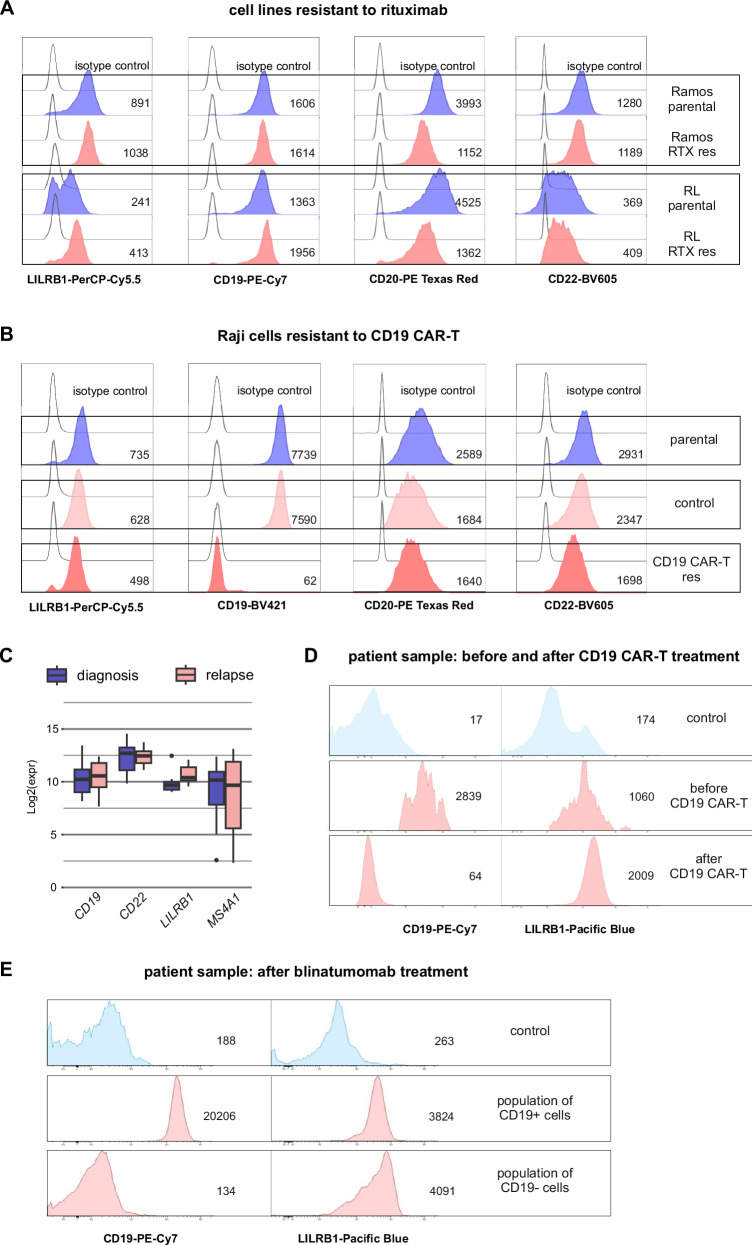
LILRB1 remains stably expressed in malignant B-cells that are resistant to prior lines of immunotherapy. **A** LILRB1 expression was evaluated by flow cytometry on B-NHL cell lines (Ramos, RL) with developed in vitro resistance to RTX^20^. The cells were stained with anti-LILRB1 Ab. MFI for each sample is shown next to the corresponding histogram. **B** LILRB1 expression was evaluated by flow cytometry on B-NHL cells (Raji) with developed in vitro resistance to CD19 CAR-T cells (CD19 CAR-T res) and compared to parental Raji cells and the cells exposed to unmodified T cells (control). The cells were stained with anti-LILRB1 Ab. MFI for each sample is shown next to the corresponding histogram. **C** Box plots showing mRNA expression levels of *LILRB1*, *CD19*, *CD22*, and *MS4A1* in eight patients with matched RNA-seq data at diagnosis and post-relapse from CD19 CAR-T therapy as described in Orlando et al. [2]. Raw counts were extracted from BAM^21^ files deposited in the SRA^22^ under accession number PRJNA451298 and normalized. **D** LILRB1 and CD19 protein levels were assessed by flow cytometry in B-ALL cells before CD19 CAR-T therapy and after relapse. Normal T and NK cells served as negative controls. The MFI values are displayed near the histograms. **E** LILRB1 and CD19 protein levels were assessed by flow cytometry in treatment-refractory B-ALL cells following one cycle of blinatumomab treatment. LILRB1 expression was evaluated on both CD19^+^ and CD19^-^ cells, with results shown on separate histograms. Normal T and NK cells served as negative controls. The MFI values are displayed near the histograms. In (**A**, **B**, **D**, **E**), clone HP-F1 of the anti-LILRB1 Ab was used.

Antigen escape or downregulation are established mechanisms of resistance to various immunotherapies. Therefore, we utilized models resistant to previous lines of immunotherapy [25] in B-NHL cell lines that were LILRB1-positive (surprisingly, LILRB1 was expressed only in 2 out of 8 analyzed B-ALL cell lines and 4 out of 6 B-NHL cell lines) (Supplementary Fig. 4A, B). In lymphoma models resistant to rituximab (RTX), an anti-CD20 antibody, LILRB1 levels tended to increase, while CD20 was downregulated, and CD22 and CD19 remained largely unchanged (Fig. 4A). We also assessed the level of selected markers in in vitro-generated lymphoma Raji cells resistant to CD19 CAR-T cells [26]. We observed a loss of CD19 expression accompanied by only partial downregulation of CD22, CD20, and LILRB1 (Fig. 4B). In RNA-seq data from B-ALL patients who lost surface CD19 following CD19 CAR-T therapy [2], post-therapy samples showed increased *LILRB1* mRNA levels, while *CD22* and *MS4A1* (codes for CD20) levels were reduced (Fig. 4C). Although *CD19* transcript levels also slightly increased (Fig. 4C), they corresponded to transcripts encoding truncated, nonfunctional CD19 as reported by Orlando et al. [2]. Next, in the retrospective analysis comparing B-ALL paired samples (before/after anti-CD19 immunotherapy), we observed loss or significant CD19 downregulation in blasts isolated from 16 patients (Supplementary Fig. 4C). Furthermore, in 10/16 cases, CD22 exhibited a concurrent decrease alongside CD19, while CD20 levels were either low or absent in most cases, already prior to immunotherapy (Supplementary Fig. 4C), demonstrating the disadvantages of these targets for next-line treatments. Notably, in our cohort of 16 patients, one patient with CD19-negative relapse after CD19 CAR-T cell therapy was examined for LILRB1 expression and demonstrated a robust increase in LILRB1 levels (Fig. 4D). Furthermore, we analyzed a sample from a patient who relapsed after blinatumomab treatment and displayed a mixed CD19^−^/^+^ phenotype. Importantly, in this sample robust LILRB1 expression was detected, with stable levels even in the CD19-negative subpopulation of leukemic cells (Fig. 4E). Altogether, these findings further reinforce the critical necessity for the identification of non-B-cell-specific targets to serve as substitutes in patients experiencing CD19-negative relapses and support LILRB1 CAR-T cells as a valid next-line treatment.

### Development of LILRB1-directed CAR-T cells

**Fig. 5:**
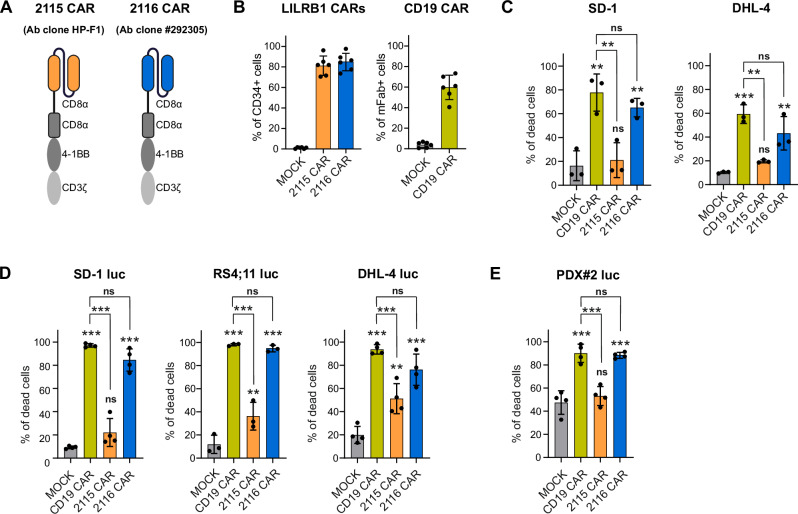
Generation of LILRB1 CAR-T cells and preliminary in vitro efficacy testing. **A** A scheme representing modular structure of generated LILRB1 CARs: LILRB1-targeting scFv^23^ sequences derived from two different clones of LILRB1 mAb^24^, HP-F1 and #292305, were incorporated into a second-generation CAR backbone comprising CD8 hinge, CD8 transmembrane domain, and 4-1BB-CD3ζ signaling tail. **B** Efficiency of primary T cells transduction with CAR constructs. The expression of LILRB1 CARs was evaluated by staining the cells with anti-CD34 Ab, CD19 CAR presence was confirmed using anti-mFab^25^ Ab. Data shows means ± SD^26^ from *n* = 6 donors. **C** Cytotoxicity of LILRB1 CAR-T cells against B-cell leukemia (SD-1) and lymphoma (DHL-4) cells was assessed by flow cytometry-based killing assay. CAR-T cells and CTV^27^-labeled target cells were co-cultured for 24 h at 1:1 E:T^28^ ratio. The samples were then stained with PI^29^, and the percentage of dead CTV^+^PI^+^ target cells was determined. Data shows mean ± SD from *n* = 3 donors, *P* values were calculated using ordinary one-way ANOVA with Tukey’s multiple comparisons test (MOCK vs. CAR-T and various CAR-T comparisons). **D** Cytotoxicity of LILRB1 CAR-T cells against B-cell leukemia (SD-1, RS4;11) and lymphoma (DHL-4) cells was assessed using a luciferase-based killing assay. CAR-T cells and luciferase-expressing target cells were co-cultured for 24 h at a 1:1 E:T ratio. The percentage of dead target cells was determined by measuring the decrease in luminescence signal. Data represent mean ± SD from *n* = 3 donors. *P* values were calculated using ordinary one-way ANOVA with Tukey’s multiple comparisons test (MOCK vs. CAR-T and various CAR-T comparisons). **E** Cytotoxicity of LILRB1 CAR-T cells against KMT2A-r B-ALL PDX cells was assessed by luciferase-based killing assay. CAR-T cells and luciferase-expressing target cells were co-cultured for 24 h at 0.5:1 E:T ratio. The percentage of dead target cells was determined by measuring the decrease in luminescence signal. Data shows mean ± SD from *n* = 3 donors, *P* values were calculated using ordinary one-way ANOVA with Tukey’s multiple comparisons test (MOCK vs. CAR-T and various CAR-T comparisons).

To generate LILRB1 CAR-T cells and select LILRB1-specific single-chain variable fragments (scFv), we screened various clones of murine anti-LILRB1 monoclonal antibodies (mAbs) in B-ALL cell lines exhibiting various levels of LILRB1, ultimately choosing two clones: HP-F1 and #292305 (Supplementary Fig. 5A). We utilized de novo antibody sequencing via LC-MS/MS to derive the antibodies variable domains. They were subsequently used to design the antigen-binding domains, the single-chain variable fragments (scFv), which were then integrated into a second-generation CAR backbone (CD8 hinge, CD8 transmembrane domain, 4-1BB-CD3z signaling), linked by 2 A ribosome-skipping peptide to a truncated CD34 protein, enabling transduced cells’ detection. The resulting CARs derived from HP-F1 and #292305 antibody clones were designated as constructs 2115 and 2116, respectively (Fig. 5A). Primary T cells from healthy donors were transduced with these CAR constructs, and the CD34 signal was observed in 70–90% of T cells, indicating robust transduction efficiency (Fig. 5B, left panel). Similarly, sufficient transduction efficiency and CAR expression were detected in CD19 CAR-T cells, as proved by high levels of murine antigen-binding fragment (Fig. 5B, right panel).

### LILRB1 CAR-T cells exhibit activity against leukemia and lymphoma cells

We evaluated the killing potential of LILRB1 CAR-T cells generated using both 2115 and 2116 constructs. As target cells, we selected representative B-ALL (SD-1) and B-NHL (DHL-4) cell lines with the highest expression of LILRB1 among tested cells (Supplementary Fig. 4A, B). In both cell lines, 2116 LILRB1 CAR-T cells, but not 2115 LILRB1 CAR-T cells, exhibited potent killing efficacy comparable to CD19 CAR-T cells, as assessed by flow cytometry (Fig. 5C) and in bioluminescence-based killing assay (Fig. 5D). Importantly, 2116 LILRB1 CAR-T effectively killed RS4;11 B-ALL cells with moderate LILRB1 levels (Fig. 5D, Supplementary Fig. 4A, 5A). Subsequently, both 2116 LILRB1 and CD19 CAR-T cells killed B-ALL PDX cells expressing luciferase with similar efficacy, while 2115 LILRB1 CAR-T cells did not exhibit any advantage over MOCK T cells (Fig. 5E). Based on these findings, we concluded that the 2116 LILRB1 CAR construct surpassed the 2115 LILRB1 construct, warranting its selection for subsequent experiments. Hereafter, the 2116 LILRB1 CAR will be referred to as LILRB1 CAR.

### LILRB1 CAR-T cells specifically kill LILRB1-expressing cells and induce potent cytokine production

**Fig. 6:**
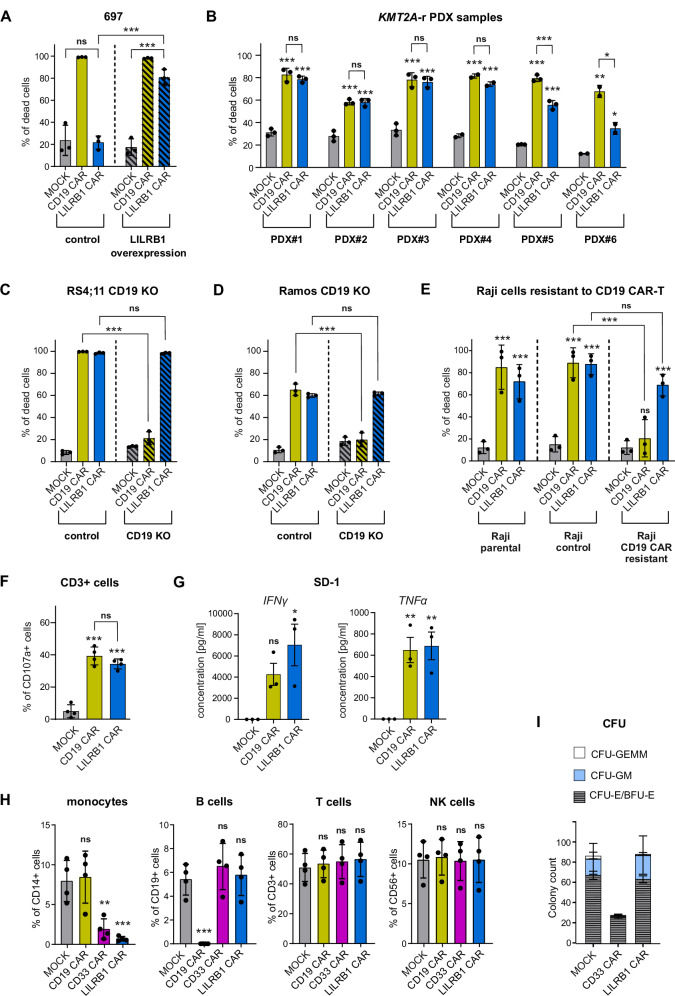
LILRB1 CAR-T cells demonstrate target specificity and functionality after antigen stimulation. **A** Cytotoxicity of LILRB1 CAR-T cells against B-ALL cells (697) genetically modified to overexpress LILRB1 was assessed by flow cytometry-based killing assay. CAR-T cells and CTV-labeled target cells were co-cultured for 24 h at 1:1 E:T ratio. The samples were then stained with PI, and the percentage of dead CTV^+^PI^+^ target cells was determined. Data shows mean ± SD from *n* = 3 donors, *P* values were calculated using two-way ANOVA with Tukey’s multiple comparisons test (MOCK vs. CAR-T cells in each group and CAR-T vs. control or modified cell line). **B** Cytotoxicity of LILRB1 CAR-T cells against 6 KMT2A-r B-ALL PDX cells was assessed by flow cytometry-based killing assay. CAR-T cells and CTV-labeled target cells were co-cultured for 24 h at 0.5:1 E:T ratio. The samples were then stained with PI, and the percentage of dead CTV^+^PI^+^ target cells was determined. Data shows mean ± SD from *n* = 2–3 donors, *P* values were calculated using ordinary one-way ANOVA with Tukey’s multiple comparisons test (MOCK vs. CAR-T and various CAR-T comparisons). **C** Cytotoxicity of LILRB1 CAR-T cells against B-ALL cells (RS4;11) with CD19 KO^30^ was assessed using a luciferase-based killing assay. CAR-T cells and luciferase-expressing target cells were co-cultured for 24 h at a 2:1 E:T ratio. The percentage of dead target cells was determined by measuring the decrease in luminescence signal. Data shows mean ± SD from *n* = 3 donors, *P* values were calculated using two-way ANOVA with Tukey’s multiple comparisons test (CAR-T vs. control or CD19 KO cell line). **D** Cytotoxicity of LILRB1 CAR-T cells against B-NHL cells (Ramos) with CD19 KO was assessed by flow cytometry-based killing assay. CAR-T cells and CTV-labeled target cells were co-cultured for 24 h at 2:1 E:T ratio. The samples were then stained with PI, and the percentage of dead CTV^+^PI^+^ target cells was determined. Data shows mean ± SD from n = 3 donors, *P* values were calculated using two-way ANOVA with Tukey’s multiple comparisons test (CAR-T vs. control or CD19 KO cell line). **E** Cytotoxicity of LILRB1 CAR-T cells against Raji cells resistant to CD19 CAR-T cells was assessed using a luciferase-based killing assay. CAR-T cells and luciferase-expressing target cells were co-cultured for 24 h at 5:1 E:T ratio. The percentage of dead target cells was determined by measuring the decrease in luminescence signal. Data shows means ± SD from *n* = 3 donors, *P* values were calculated using two-way ANOVA with Tukey’s multiple comparisons test (MOCK vs. CAR-T cells in each group and CAR-T vs. control or resistant cell line). **F** Degranulation of LILRB1 CAR-T cells was assessed by flow cytometry. CAR-T cells and target cells (SD-1) were co-incubated for 18 h at 1:2 E:T ratio, in the presence of anti-CD107a Ab. Next, the samples were stained with anti-CD3 Ab, and the percentage of CD3^+^CD107a^+^ T cells was determined. Data shows mean ± SD from *n* = 4 donors, *P* values were calculated using ordinary one-way ANOVA with Tukey’s multiple comparisons test (MOCK vs. CAR-T and CAR-T comparison). **G**. Cytokine release by LILRB1 CAR-T cells was evaluated using ELISA^31^ assay. CAR-T cells and target cells (SD-1) were co-incubated for 24 h at 1:2 E:T ratio. The concentration of IFNγ^32^ and TNFα^33^ was then measured in the culture medium. Data shows mean ± SEM^34^ from *n* = 3 donors, *P* values were calculated using ordinary one-way ANOVA with Dunnett’s multiple comparisons test (MOCK vs. CAR-T). **H** Cytotoxicity of LILRB1 CAR-T cells against normal PBMC^35^ was assessed by flow cytometry. CFSE^36^-labeled CAR-T cells were co-incubated with PBMCs for 24 h at 1:2 E:T ratio. The samples were then stained with anti-CD19, anti-CD56, anti-CD3, and anti-CD14 antibodies to determine the approximate percentages of B-cells (CD19^+^ cells), monocytes (CD14^+^ cells), T cells (CD3^+^ cells), and NK cells (CD56^+^ cells). Data shows mean ± SD from n = 4 donors, *P* values were calculated using ordinary one-way ANOVA with Dunnett’s multiple comparisons test (MOCK vs. CAR-T). **I** CFU-E^37^, BFU-E^38^, CFU-GM^39^, and CFU-GEMM^40^ colonies were counted upon co-culture of 2.0 × 10^5^ BMNC^41^ with 2.0 × 10^6^ CAR-T cells respectively (E:T ratio of 10:1) for 6 h, followed by plating in methylcellulose and incubated for 14 days at 37 °C (*n* = 3; technical replicates).

To verify the specificity of LILRB1 CAR-T cells, we overexpressed LILRB1 in 697 cells (B-ALL) that were originally LILRB1-negative (Supplementary Fig. 5B). LILRB1 CAR-T cell killing activity was exclusively detected in 697 cells overexpressing LILRB1 (Fig. 6A). Furthermore, LILRB1 CAR-T cells exhibited no cytotoxic effects against LILRB1-negative B-NHL cells (Supplementary Fig. 5C) as well as non-hematologic LILRB1-negative cells, such as breast and hepatocellular carcinoma cell lines (Supplementary Fig. 5D, E). Subsequently, we compared the cytotoxic effects of LILRB1 CAR-T cells with CD19 CAR-T cells against KMT2A-r B-ALL PDX with varying LILRB1 levels. LILRB1 CAR-T cells exhibited cytotoxicity against four PDX samples (PDX#1-PDX#4), which was comparable to CD19 CAR-T cells cytotoxicity (Fig. 6B), consistent with their high LILRB1 and CD19 levels (Supplementary Fig. 5F). Conversely, lower cytotoxicity of LILRB1 CAR-T cells as compared to CD19 CAR-T cells was observed in samples PDX#5 and PDX#6 (Fig. 6B), which expressed low LILRB1 levels (Supplementary Fig. 5F). To evaluate the potential of LILRB1 CAR-T cells in targeting CD19-negative relapses, we developed CD19 knockout (CD19 KO) cell line models. Our analysis revealed that LILRB1 expression remained stable in CD19-negative cells (Supplementary Fig. 5G). While CD19 KO cells exhibited complete resistance to CD19 CAR therapy, they were effectively eliminated by LILRB1 CAR-T cells (Fig. 6C, D). Furthermore, LILRB1 CAR-T cells potently killed Raji cells resistant to CD19 CAR-T cells (Fig. 6E) that aligned with the preserved LILRB1 expression (Fig. 4B). Altogether, our findings confirm the specificity of LILRB1 CAR-T cells and demonstrate that their cytotoxicity corresponds with LILRB1 levels on target cells.

To validate the functionality of generated LILRB1 CAR-T cells, we tested their degranulation in the presence of cells expressing high LILRB1 levels (SD-1). LILRB1 CAR-T cells exhibited pronounced degranulation, comparable to CD19 CAR-T cells (Fig. 6F), and released significant amounts of IFNγ and TNFα (Fig. 6G). These findings collectively demonstrate target-specific degranulation and cytokine release by LILRB1 CAR-T cells.

### LILRB1 CAR-T cells exhibit minimal toxicity against normal leukocytes apart from monocytes

To assess potential off-tumor toxicity across normal leukocytes, we conducted co-culture experiments with LILRB1 CAR-T cells and healthy donor peripheral blood mononuclear cells (PBMC). Flow cytometry analysis of PBMC subtypes revealed specific depletion of monocytes by LILRB1 CAR-T cells, with no discernible effects on B cells, T cells or NK cells (Fig. 6H, Supplementary Fig. 6). Monocyte depletion was also observed for CD33 CAR-T cells, a therapy currently tested in clinical trials in AML patients [27]. In contrast, CD19 CAR-T cells selectively eliminated CD19-expressing B cells (Fig. 6H, Supplementary Fig. 6). We next assessed the toxicity of LILRB1 CAR-T cells on normal hematopoiesis in comparison to CD33 CAR-T cells. CD33 CAR-T cells significantly reduced the number of colonies derived from erythroid and myeloid progenitors, whereas bone marrow treated with LILRB1 CAR-T cells produced a similar number of colonies as the MOCK-treated group (Fig. 6I). These findings suggest that targeting LILRB1 is likely to spare normal leukocytes, except for monocytes, and that LILRB1 CAR-T cells are less myelotoxic than CD33 CAR-T cells, which remains in accordance with LILRB1 expression profile (Fig. 2A, C).

### LILRB1 CAR-T cells demonstrate anti-B-ALL activity in vivo

**Fig. 7:**
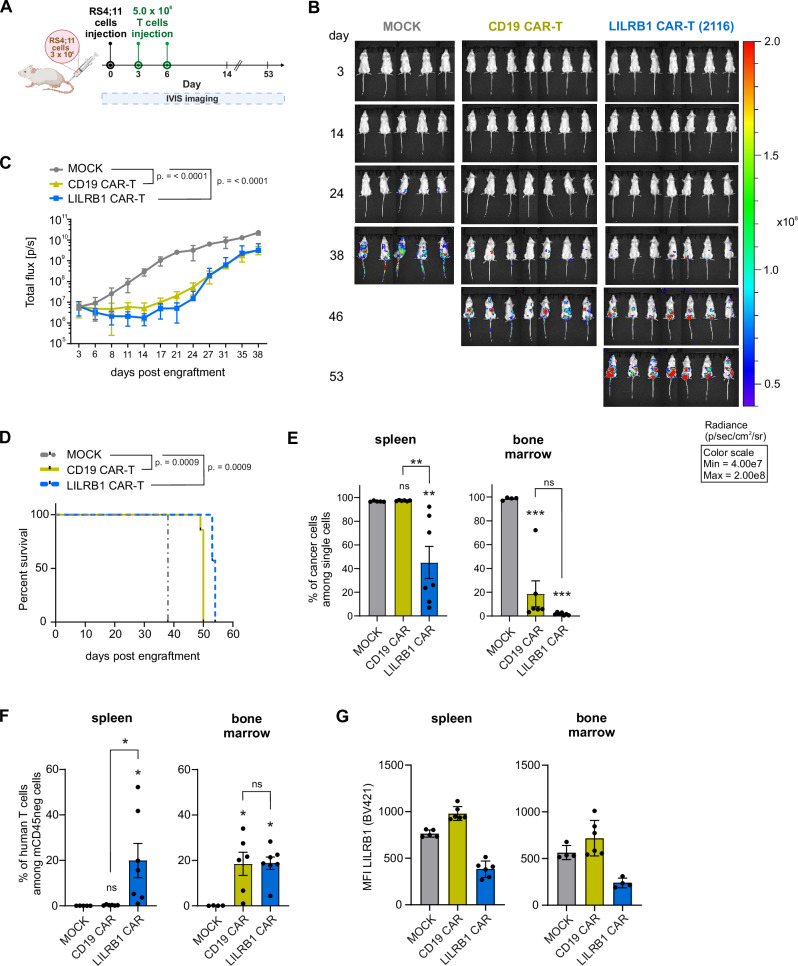
LILRB1 CAR-T cells exhibit in vivo efficacy against LILRB1^+^ B-ALL cells*.* **A** A scheme representing the design of the experiment evaluating the in vivo efficacy of LILRB1 CAR-T cells. NSG^42^ mice were injected *iv*^43^ with 3.0 × 10^6^ of luciferase-expressing RS4;11 cells. On days 3 and 6 after the injection of cancer cells, the mice were treated with two doses, each of 5.0 × 10^6^ of CAR-T cells. The tumor development was monitored using IVIS^44^ imaging system. **B** Representative images of the mice with developing tumors were obtained from the IVIS imaging system on days 3, 14, 24, 38, 46, and 53 following RS4;11 cells injection. The radiance scale demonstrates bioluminescence intensity. **C** The quantification of tumor development was performed based on the measured bioluminescence from a region of interest drawn over each animal and is presented as the mean ± SD of total flux signal (*n* = 5–7/group) over time. *P* values were calculated for the results obtained up to day 38 using two-way repeated measures ANOVA with Dunnett’s multiple comparisons test (MOCK vs. CAR-T). The *P* values on day 38 are displayed on the graph. **D** Event-free survival depicted on Kaplan–Meier survival plot. Curve comparison was done by log-rank (Mantel–Cox) test. **E** The percentage of RS4;11 cells among all analyzed cells present in the spleens and bone marrow of the mice following the treatment was determined based on flow cytometry analysis at the day of sacrifice. Human cancer cells were defined as cells negative for murine CD45 (mCD45) antigen and human CD3 antigen. Data shows mean ± SEM from *n* = 4–7 mice, *P* values were calculated using ordinary one-way ANOVA with Tuckey’s multiple comparisons test. **F** The percentage of human T cells residing in the spleens and bone marrow of the mice following CAR-T treatment was determined based on flow cytometry analysis. Human CD3^+^ cells were gated out from the cell population negative for murine CD45 antigen. Data shows mean ± SEM from *n* = 4–7 mice, *P* values were calculated using ordinary one-way ANOVA with Tuckey’s multiple comparisons test. **G** LILRB1 surface expression on the RS4;11 cells obtained from spleens and bone marrow of the mice following CAR-T treatment was assessed by flow cytometry.

To assess the in vivo efficacy of the LILRB1 CAR, we utilized NOD scid gamma (NSG) mice implanted with RS4;11 B-ALL cells expressing GFP-luciferase (GFP-luc+) (Fig. 7A). Mice treated with two doses of either LILRB1 CAR-T cells or CD19 CAR-T cells exhibited a significant reduction in tumor burden, as measured by bioluminescent imaging (Fig. 7B, C), and had markedly prolonged survival compared to those receiving MOCK T cells (Fig. 7D). At the time of sacrifice, organ analysis showed that mice treated with LILRB1 CAR-T cells had significantly lower tumor burden in the spleens compared to the groups after MOCK and CD19 CAR-T cells, and significantly lower tumor burden in the bone marrow compared to the group treated with MOCK T cells (Fig. 7E, Supplementary Fig. 7). Additionally, a significant number of human CD3^+^ cells were detected among human cells in the spleens and bone marrow of the LILRB1 CAR-T cell-treated group (Fig. 7F). Importantly, RS4;11 tumor cells that relapsed following CD19 CAR-T treatment continued to express LILRB1 (Fig. 7G). These findings indicate that LILRB1 CAR-T cells are effective against B-ALL in vivo and their antitumor efficacy is comparable to that of CD19 CAR-T cells.

### LILRB1 is expressed in monocytic AML, and LILRB1 CAR-T cells eliminate monocytic AML both in vitro and in vivo

**Fig. 8:**
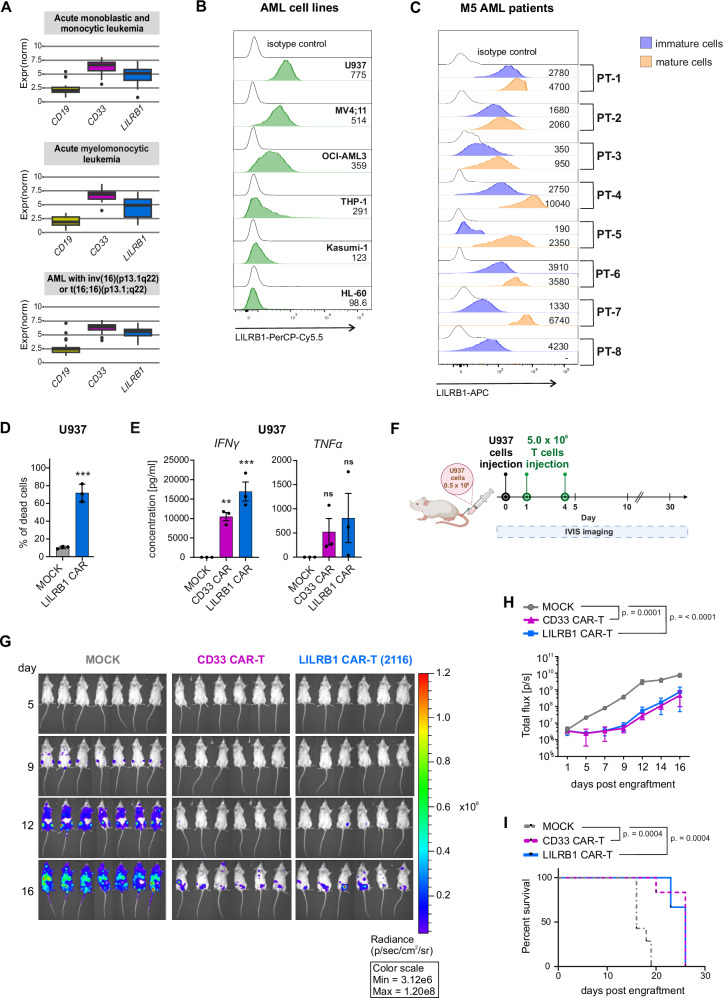
LILRB1 CAR-T cells exhibit in vitro and in vivo efficacy against LILRB1^+^ monocytic AML^45^. **A** Box plots showing *LILRB1*, *CD19* (negative control), and *CD33* (positive control) mRNA expression in various subtypes of AML from BeatAML2 dataset [28]. Normalized expression values were downloaded from https://biodev.github.io/BeatAML2/. **B** LILRB1 expression was evaluated by flow cytometry on various AML cell lines. The cells were stained with anti-LILRB1 Ab (clone HP-F1). MFI for each cell line is shown next to the corresponding histogram. **C**. LILRB1 expression was evaluated by flow cytometry using anti-LILRB1 Ab (clone HP-F1) on samples obtained from M5 AML patients. Total monocytoid cells population was selected on CD64 *vs* CD36 plot. Pathological cells were further divided based on characteristic immunophenotype pattern of immature monocytoid cells: CD13^+dim^CD14^(−)^HLA-DR^+^CD15^+^CD11b^+dim^ and mature monocytoid cells/monocytes: CD13^+^CD14^+^HLA-DR^+^CD15^(−)^CD11b^+^. LILRB1 expression is presented for these two populations of gated malignant cells. MFI for each sample is shown next to the corresponding histogram. **D** Cytotoxicity of LILRB1 CAR-T cells against AML cells (U937) was assessed using a flow cytometry-based killing assay. CAR-T cells and CTV-labeled target cells were co-cultured for 24 h at 1:1 E:T ratio. The samples were then stained with PI, and the percentage of dead CTV^+^PI^+^ target cells was determined. Data shows mean ± SD from *n* = 3 donors, *P* values were calculated using a t-test (MOCK vs. CAR-T). **E** IFNγ and TNFα release by LILRB1 CAR-T cells was evaluated using ELISA assay. CAR-T cells and target AML cells (U937) were co-incubated for 24 h at 1:2 E:T ratio. The concentration of IFNγ and TNFα was then measured in the culture medium. Data shows mean ± SEM from *n* = 3 donors, *P* values were calculated using ordinary one-way ANOVA with Dunnett’s multiple comparisons test (MOCK vs. CAR-T). **F** A scheme representing design of the experiment evaluating in vivo efficacy of LILRB1 CAR-T cells. NSG mice were injected *iv* with 0.5 × 10^6^ of luciferase-expressing U937 cells. On days 1 and 4 after the injection of cancer cells, the mice were treated with two doses, each of 5.0 × 10^6^ of CAR-T cells. The tumor development was monitored using IVIS imaging system. **G**. Representative images of mice with developing tumor were obtained from the IVIS imaging system on days 5, 9, 12, and 16, following U937 cells injection. The radiance scale demonstrates bioluminescence intensity. **H** The quantification of tumor development was performed based on the measured bioluminescence from a region of interest drawn over each animal and is presented as the mean ± SD of total flux signal (*n* = 6–7/group) over time. *P* values were calculated for the results obtained up to day 16 using two-way repeated measures ANOVA with Dunnett’s multiple comparisons test (MOCK vs. CAR-T). The *P* values on day 16 are displayed on the graph. **I** Event-free survival depicted on Kaplan–Meier survival plot. Curve comparison was done by log-rank (Mantel–Cox) test.

Considering the abundant expression of LILRB1 in monocytes, we also checked *LILRB1* mRNA levels in various AML subtypes from the BeatAML2 study [28] deposited in the Genotype and Phenotype (dbGaP) database (study phs001657.v3.p1). We found *LILRB1* expression generally lower than CD33 across subtypes, with the highest expression observed in monocytic/monoblastic/myelomonocytic AML and AML with chromosome 6 abnormalities (Fig. 8A, Supplementary Fig. 8A). Next, we evaluated LILRB1 surface protein levels in AML cell lines and monocytic AML primary cells. Consistent with mRNA data, the highest levels of LILRB1 protein were found in monocytic (U937) and bi-phenotypic B-myelomonocytic (MV4;11) cell lines (Fig. 8B) as well as in M5 AML primary cells (Fig. 8C, Supplementary Table 6). Accordingly, we observed potent in vitro killing of LILRB1-expressing AML cell line (U937) by LILRB1 CAR-T cells (Fig. 8D) and prominent cytokine release (Fig. 8E).

Finally, we investigated the in vivo efficacy of LILRB1 CAR-T cells in AML using an aggressive model of U937 cells in NSG mice. Mice treated with two doses of either LILRB1 CAR-T cells or CD33 CAR-T cells (Fig. 8F) exhibited a robust response, as evidenced by reduced leukemic burden observed through bioluminescent imaging (Fig. 8G, H) and significantly prolonged survival compared to MOCK T cells treatment (Fig. 8I). To assess LILRB1 levels on cancer cells post-CAR treatment, we isolated spleens from the mice at the time of sacrifice. Although LILRB1 levels in U937 cells were much lower in vivo than in vitro, they were stable across treatment groups (Supplementary Fig. 8B, C). Collectively, these results show that LILRB1 CAR-T cells effectively reduce leukemic burden and prolong survival in this aggressive AML model.

## Discussion

In this study, we identified LILRB1 as a novel target for CAR-T cell immunotherapy of hematologic malignancies. LILRB1, primarily expressed in lymphoid tissue, belongs to the group of immune inhibitory receptors that bind MHC class I molecules to transmit inhibitory signals [29, 30]. Blocking antibodies targeting LILRB1-mediated signaling have previously been shown to enhance NK cell antitumor activity and tumor cell killing [30, 31]. Herein, we demonstrate that LILRB1 is expressed in various hematological malignancies, including B-ALL, B-NHL, as well as monocytic AML. Previous studies have also reported LILRB1 expression in B-ALL and monocytes [32, 33]. Notably, LILRB1 shows minimal expression outside the lymphoid compartment, enhancing its suitability as an immunotherapy target. Herein, we present that CAR-T cells targeted against LILRB1 specifically kill LILRB1-expressing tumor cells both in vitro and in vivo while sparing LILRB1-negative cells.

LILRB1 expression profile in normal tissues is suitable for targeting by CAR. It is minimally expressed in activated T cells and CAR-T cells, thus enabling efficient CAR-T cell generation. Furthermore, it is expressed at low levels on HSCs, HSPCs, and mature granulocytes, potentially reducing the risk of treatment-related immunosuppression. While hematopoietic cell transplantation (HSCT) is a recognized cure for myelotoxicity, it carries significant risks. The need for HSCT post-CAR-T therapy varies by disease type, largely due to the differences in the distribution of antigens targeted by CARs. In B-ALL and B-NHL, post- CAR-T HSCT can be beneficial but also poses serious toxicity risks, particularly in adult patients, therefore, it is most often considered after CAR-T treatment failure [34, 35]. In AML, CD33 CAR-T cells target HSPCs and CD33-expressing neutrophils, leading to marrow hypoplasia and severe neutropenia, often making HSCT necessary [36]. Emerging strategies, such as drug-regulated CD33 CARs [37] or combinatorial approaches [38], show promise in reducing HSPC toxicity. LILRB1 CAR-T cells offer a distinct advantage by sparing HSCs and neutrophils, potentially avoiding severe immunosuppression and providing a safer alternative to currently tested CAR-T therapies in AML.

In this study, we constructed two different LILRB1-specific CAR molecules, 2115 and 2116, based on two clones of anti-LILRB1 mAbs: HP-F1 and #292305, respectively. T cells expressing the 2116 CAR construct were significantly more efficient in killing LILRB1-expressing cells. Given the substantial differences in the variable regions of the heavy chains of these two antibody clones (data not shown), we speculate that they bind different LILRB1 epitopes. In accordance with previously published work, this may affect the immune synapse structure and, consequently, CAR efficacy [39]. Focusing on the more efficient 2116 variant, we conducted extensive preclinical validation of the LILRB1 CAR-T cells both in vitro and in vivo. Based on our results, we demonstrate for the first time that LILRB1 is a viable target for immunotherapy and propose LILRB1 CAR-T cells as a prototype for a novel strategy applicable across various hematologic malignancies.

To assess the applicability of LILRB1 CAR-T cells as a salvage therapy, we employed cell lines resistant to previous treatments. Since rituximab plus chemotherapy (R-CHOP) is a first-line treatment for lymphoma, we used two distinct RTX-resistant cell lines. Notably, these cell lines showed upregulation of LILRB1 compared to controls, supporting LILRB1’s utility as a target in RTX-resistant patients. Furthermore, given that CD19-directed immunotherapy is effective long-term only in about 50% of patients, we used a CD19 CAR-T-resistant cell line generated by exposing it to CD19 CAR-T cells over multiple cycles, mirroring clinical data where resistance may result from CD19 loss. In this model, CD19 loss was accompanied by disturbances in pathways related to lymphocyte proliferation, activation, and adhesion [26], while LILRB1 expression remained stable. Importantly, LILRB1 expression was preserved in primary cells from patients with CD19-negative relapses after previous CD19-targeted therapies. Our findings align with a recent study identifying LILRB1 as a marker of CD19-negative B-ALL lymphoblasts that relapsed after CD19 CAR-T therapy [40]. Moreover, our retrospective analysis of patients previously treated with CD19-targeting immunotherapy revealed that CD22 is often lost or downregulated along with CD19, as reported before [11], while CD20 is typically expressed at low levels. These observations suggest that CD22 and CD20 may not always be optimal targets. Therefore, LILRB1 CAR-T cells could be particularly beneficial for patients resistant to previous treatments, providing an alternative to other CAR-T cells tested in preclinical studies, targeting CD72, CD79a, or CD79b [7, 8, 41].

LILRB1 CAR-T cells may also be useful for treating B-ALL cases with a lymphoid-to-myeloid lineage switch. Although rare in pediatric B-ALL cases treated with chemotherapy [42], lineage switch or myeloid feature acquisition occurs in up to 10% of patients treated with CD19-directed immunotherapy [13, 14]. B-ALL cells, originating from committed pre-B cells or earlier progenitors, can reprogram into other hematopoietic lineages, often adopting a monocytic or myelomonocytic phenotype [14]. Prolonged CD19-directed immunotherapy can induce lineage switching, characterized by loss of B-cell markers (CD19, CD22, B220) and appearance of myeloid markers (CD33, Gr1, CD11b) [43]. Instances of myeloid lineage switching have been documented following blinatumomab and CD19 CAR-T treatments [44–46]. In these scenarios, LILRB1 CAR-T cells could offer a valuable alternative to already existing therapies.

LILRB1 CAR-T cells also offer a promising treatment option for monocytic and mixed-lineage AML. Our findings show that LILRB1 is highly expressed in normal monocytes and monocytic AML primary cells, and LILRB1 CAR-T cells effectively eliminate these cells in preclinical in vitro and in vivo models. Monocytic AML (M5 by FAB classification) is a difficult-to-treat, life-threatening malignancy, constituting about 10% of AML cases. M5 AML cells exhibit increased resistance to venetoclax, with relapses showing a monocytic phenotype in around 30% of patients, resulting in reduced overall survival compared to non-M5 AML cases [47]. Given the need for effective immunotherapy targets for monocytic AML, several candidates such as CD64 [48], LILRB4 [49], and CLL1 [50] have been preliminarily evaluated. However, it should be highlighted that no sustainable efficacy in patients has been demonstrated so far with any of the currently tested CAR-T therapies in AML [18]. Although still in early stages of development, LILRB1 CAR-T therapy represents a promising alternative to existing strategies to be tested in further preclinical studies and clinical trials.

Finally, as LILRB1 is expressed in monocytes and macrophages, both broader applicability and potential toxicities of LILRB1 CAR-T cells can be anticipated. On one hand, eliminating non-classical monocytes and tumor-associated macrophages could enhance the efficacy of other cancer therapies, particularly in tumors with monocyte/macrophages accumulation, which is linked to poor survival [51–53]. This approach is being explored in clinical trials targeting macrophage receptor CSF1R [54]. On the other hand, LILRB1 expression on Kupffer cells raises concerns about hepatic toxicity, similarly to the risks associated with CD33-targeting therapies. CD33 is also expressed on Kupffer cells, and severe hepatic toxicity, including veno-occlusive disease, has been reported with CD33-targeting antibody-drug conjugates, but this toxicity was attributed to the calicheamicin component rather than the targeting of Kupffer cells [55]. Importantly, such toxicity has not been observed in early-phase clinical trials of CD33 CAR-T cells [27, 36]. Nonetheless, potential hepatic toxicity with LILRB1 CAR-T therapy remains a concern and warrants further investigation in preclinical models.

In conclusion, we have developed LILRB1 CAR-T cells effective against B-ALL, B-NHL, and monocytic AML. Our research highlights the potential of LILRB1 CAR-T cells to overcome the limitations of current immunotherapies, particularly for patients resistant to CD19-directed treatments or those with monocytic AML, which currently lack CAR-T options. We identified an effective scFv in the standard 41BB-CD3ζ CAR format. Future efforts will focus on refining the scFv and exploring its integration into alternative CAR backbones to enhance therapeutic efficacy. Furthermore, scaling up LILRB1 CAR-T cell production will be a critical step in advancing toward first-in-human clinical trials.

## Materials and methods

### Cell surface protein labeling

PDX generation is described in the Supplementary Methods. Prior to cell surface protein labeling of B-ALL PDX cells, red blood cell lysis was performed on splenocyte suspensions using ACK Lysing Buffer (Thermo Fisher Scientific, cat. no. A1049201). The samples used for subsequent steps contained at least 90% of human cells. Next, 90 × 10^6^ cells were washed twice with ice-cold PBS and incubated with 0.5 mg/ml EZ-Link™ Sulfo-NHS-LC-Biotin (Thermo Fisher Scientific, cat. no. 21335) in ice-cold PBS on a rocking platform for 2 h at 4 °C. Non-biotinylated control cells were incubated with PBS under the same conditions. After centrifugation at 300 × *g* for 5 min, the pellet was resuspended in 100 mM glycine to quench the biotinylating reaction. Next, the cells were washed twice with ice-cold PBS, pelleted, and lysed in the Lysis buffer (2% NP-40, 1% Triton X-100, 10% glycerol in PBS) containing EDTA-free protease inhibitors (Roche, cat. no. 04693159001) for 30 min on ice with intermittent vortexing. The cell lysates were centrifuged at 10,000 × *g* for 2 min at 4 °C, the supernatant was transferred to a new tube and used for purification of biotinylated proteins on NeutrAvidin Agarose resin (Thermo Fisher Scientific, cat. no. 29201). For each PDX, the biotinylated samples for MS analysis were prepared in 4 technical replicates, while non-biotinylated control samples were prepared in duplicates. Before use, 75 µl of NeutrAvidin Agarose resin was washed twice with the Lysis buffer. The clarified supernatant containing 300 µg of protein was added to the resin and incubated on a rocking platform for 2 h at RT. Unbound proteins were removed by centrifugation for 1 min at 1000 × *g* and repetitive washing: 3 times with 800 µL of Lysis Buffer, then 5 times with 800 µL of Wash Buffer (100 mM Tris-HCl pH 8 in high-grade ultrapure H_2_O), then 5 times with 800 µL of 100 mM ammonium bicarbonate (ABC) in high-grade ultrapure H_2_O.

### CAR constructs

The cloning of CAR constructs was performed as previously described [56]. Briefly, sequencing of two mouse anti-LILRB1 antibodies (clone HP-F1 and clone #292305) was performed by Rapid Novor (Kitchener, Ontario, Canada). Two anti-LILRB1 scFv were ordered as DNA fragments (Eurofins) encoding for the variable domains of the heavy and light chains joined by linkers. Anti-LILRB1 scFv DNAs were cloned into a vector containing a CD8 hinge, a CD8 transmembrane domain, a 41BB co-signaling, and a CD3ζ signaling domain, linked by a 2 A ribosome skipping peptide to a truncated CD34 protein to enable verification of transduction efficiency [57]. LILRB1 CARs were expressed from a pMP71 retroviral vector. Anti-CD19 (fmc63-based) and anti-CD33 scFv-containing CAR constructs’ design was already described [57]. Overall, both constructs hold the same structure as LILRB1 CAR with CD8 hinge, CD8 transmembrane, 41BB co-signaling, and CD3ζ signaling domains.

### Animal studies testing CAR-T cells activity

All performed animal experiments complied with the EU Directive 2010/63/EU and the Polish legislation for animal experiments of the Polish Ministry of Science and Higher Education (February 26, 2015). The 2nd Local Ethical Committee for Experiments on Animals in Warsaw accessed the project and approved the use of animals in this study (WAW2/042/2023 and WAW2/077/2024). Pre-defined exclusion criteria were applied according to the ARRIVE guidelines. The NOD.Cg-*Prkdc*^*scid*^ *Il2rg*^*tm1Wjl*^/SzJ (NSG) mice (Charles River Laboratories, Wilmington, MA, USA) were in-house bred and maintained in a controlled specific pathogen-free animal facility with IVC systems. All experiments were performed using female mice aged 8–12 weeks. The sample size was calculated using the resource equation approach [58] or power analysis. For the evaluation of CAR-T cells efficacy against B-ALL, each mouse was injected via tail vein with 3 × 10^6^ RS4;11 cells stably expressing luciferase (RS4;11-luc). In vivo, bioluminescence was evaluated three days after cancer cells engraftment, and the mice were randomly allocated to control and treatment groups (5–7 mice per group). On the same day, the mice were injected via tail vein with 5.0 × 10^6^ unmodified T cells (MOCK), CD19 CAR-T cells, or LILRB1 CAR-T cells depending on the group. The second dose of MOCK or CAR-T cells was administered on day 6 post-engaftment. The in vivo imaging was performed twice a week. For the evaluation of CAR-T cells efficacy against AML, the mice were injected via tail vein with 0.5 × 10^6^ U937 cells stably expressing luciferase (U937-luc). In vivo bioluminescence was evaluated one day after cancer cells engraftment, and the mice were randomly allocated to control and treatment groups (6–7 mice per group). On the same day, depending on the group, the mice were injected with 5.0 × 10^6^ MOCK T cells, CD19 CAR-T cells, or LILRB1 CAR-T cells via the tail vein. The second dose of MOCK or CAR-T cells was administered on day 4 post engraftment. No blinding was applied during the outcome assessment (analysis of bioluminescence). The in vivo imaging was performed three times per week. In both experiments, in addition to bioluminescence detection, the mice were controlled for other signs of illness, including body weight loss, ruffled fur, hind paw reflex loss, reduced movement, and lethargy. The mice were sacrificed when they reached predefined humane endpoint criteria. The organs (spleens and bone marrow) were collected and analyzed on the same day.

The detailed description of other methods is provided in Supplementary Information.

## Supplementary information

Supplementary material

Supplementary Tables

## Data Availability

Proteomic MS data have been deposited to the PRIDE database and will be accessible upon manuscript publication under the accession number PXD058992. Data files will be made available upon reasonable request to the corresponding authors.

## Footnotes

1 B-cell Acute Lymphoblastic Leukemia

2 Patient-Derived Xenograft

3 Chimeric Antigen Receptor

4 Mass Spectrometry

5 Mean Fluorescence Intensity

6 Antibody

7 White Blood Cell

8 Bone Marrow

9 Hematopoietic Stem Cell

10 Granulocyte Colony-Stimulating Factor

11 Forward Scatter Height/ Forward Scatter Area

12 Forward Scatter Area/ Side Scatter Area

13 Fluorescence Minus One

14 European Genome-Phenome Archive

15 Gene Expression Omnibus

16 Diffuse Large B Cell Lymphoma

17 Mantle Cell Lymphoma

18 Follicular Lymphoma

19 B-Cell Non-Hodgkin Lymphoma

20 Rituximab

21 Binary Alignment Map

22 Short Reads Archive

23 Single-Chain Fragment Variable

24 Monoclonal Antibody

25 Murine Antigen Binding Fragment

26 Standard Deviation

27 Cell Trace™ Violet

28 Effector : Target

29 Propidium Iodide

30 Knockout

31 Enzyme Linked Immunosorbent Assay

32 Interferon Gamma

33 Tumor Necrosis Factor Alpha

34 Standard Error Of The Mean

35 Primary Peripheral Blood Mononuclear Cell

36 Cell trace™ Carboxyfluorescein Succinimidyl Ester

37 Colony-Forming Unit–Erythroid

38 Burst-Forming Unit–Erythroid

39 Colony-Forming Unit–Granulocyte, Macrophage

40 Colony-Forming Unit–Granulocyte, Erythrocyte, Monocyte, Megakaryocyte

41 Bone Marrow Mononuclear Cell

42 Nonobese Diabetic (NOD) Severe Combined Immunodeficiency (SCID) Gamma Mouse

43 Intravenously

44 In-Vivo Imaging System

45 Acute Myeloid Leukemia
