# Supplementary material for: LILRB1-directed CAR-T cells for the treatment of hematological malignancies
Source: Leukemia. 2025 Apr 5;39(6):1395–411. doi: 10.1038/s41375-025-02580-z (PMC12133591; doi:10.1038/s41375-025-02580-z)
Supplement: Supplementary file 1 — Supplementary material [file 41375_2025_2580_MOESM1_ESM.docx]

**LILRB1-directed CAR-T cells for the treatment of hematological malignancies**

Running head: LILRB1 as a new target for CAR-T immunotherapy

Katsiaryna Marhelava¹^,^²*, Klaudyna Fidyt²^,^³*, Monika Pepek², Marta Krawczyk¹^,^²^,^⁴, Christopher Forcados⁵^,^⁶, Agata Malinowska⁷, Bianka Swiderska⁷, Narcis Fernandez-Fuentes³, Natalia Czerwik², Iwona Baranowska¹, Agnieszka Krzywdzinska⁸, Lukasz Sedek⁹, Lukasz Slota¹⁰, Bartosz Perkowski¹⁰, Alicia Villatoro⁵, Thibault Leray⁵^,^⁶, Ewa Lech-Maranda¹¹, Pablo Menendez³^,^¹²^,^¹³^,^¹⁴^,^¹⁵^,^¹⁶, Else Marit Inderberg⁵, Sébastien Wälchli⁵, Magdalena Winiarska¹^,^² *^#^, Malgorzata Firczuk¹^,^² *^#^

¹Department of Immunology, Mossakowski Medical Research Institute, Polish Academy of Sciences, Warsaw, Poland

²Department of Immunology, Medical University of Warsaw, Warsaw, Poland

³Josep Carreras Leukemia Research Institute, Barcelona, Spain

⁴Doctoral School of Translational Medicine, Mossakowski Medical Research Institute, Polish Academy of Sciences, Centre of Postgraduate Medical Education, Warsaw, Poland

⁵Translational Research Unit, Section of Cellular Therapy, Department of Oncology, Oslo University Hospital, Oslo, Norway

⁶Institute of Clinical Medicine, Faculty of Medicine, University of Oslo, Oslo, Norway

⁷Mass Spectrometry Laboratory, Institute of Biochemistry and Biophysics, Polish Academy of Sciences, Warsaw, Poland

⁸Laboratory of Immunophenotyping, Institute of Hematology and Transfusion Medicine, Warsaw, Poland

⁹Department of Microbiology and Immunology, Medical University of Silesia in Katowice, Zabrze, Poland

¹⁰Department of Pediatric Hematology and Oncology, Medical University of Silesia in Katowice, Zabrze, Poland

¹¹Department of Hematology, Institute of Hematology and Transfusion Medicine, Warsaw, Poland

¹²Centro de Investigación Biomédica en Red-Oncología, Instituto de Salud Carlos III, Madrid, Spain

¹³Red Española de Terapias Avanzadas (TERAV), Instituto de Salud Carlos III, Madrid, Spain

¹⁴Institució Catalana de Recerca i Estudis Avançats (ICREA), Barcelona, Spain

¹⁵Department of Biomedicine, School of Medicine, University of Barcelona, Barcelona, Spain

¹⁶Institut de Recerca Hospital Sant Joan de Déu–Pediatric Cancer Center Barcelona (SJD-PCCB), Barcelona, Spain

*These authors contributed equally to this work: Katsiaryna Marhelava, Klaudyna Fidyt, Magdalena Winiarska, Malgorzata Firczuk

#Corresponding authors: Małgorzata Firczuk, email: mfirczuk@imdik.pan.pl, Department of Immunology, Mossakowski Medical Research Institute Polish Academy of Sciences, Magdalena Winiarska, email: mwiniarska@imdik.pan.pl, Department of Immunology, Mossakowski Medical Research Institute Polish Academy of Sciences

**Description: This file contains Supplementary Figures and Supplementary Materials and Methods**

**Supplementary Figures**


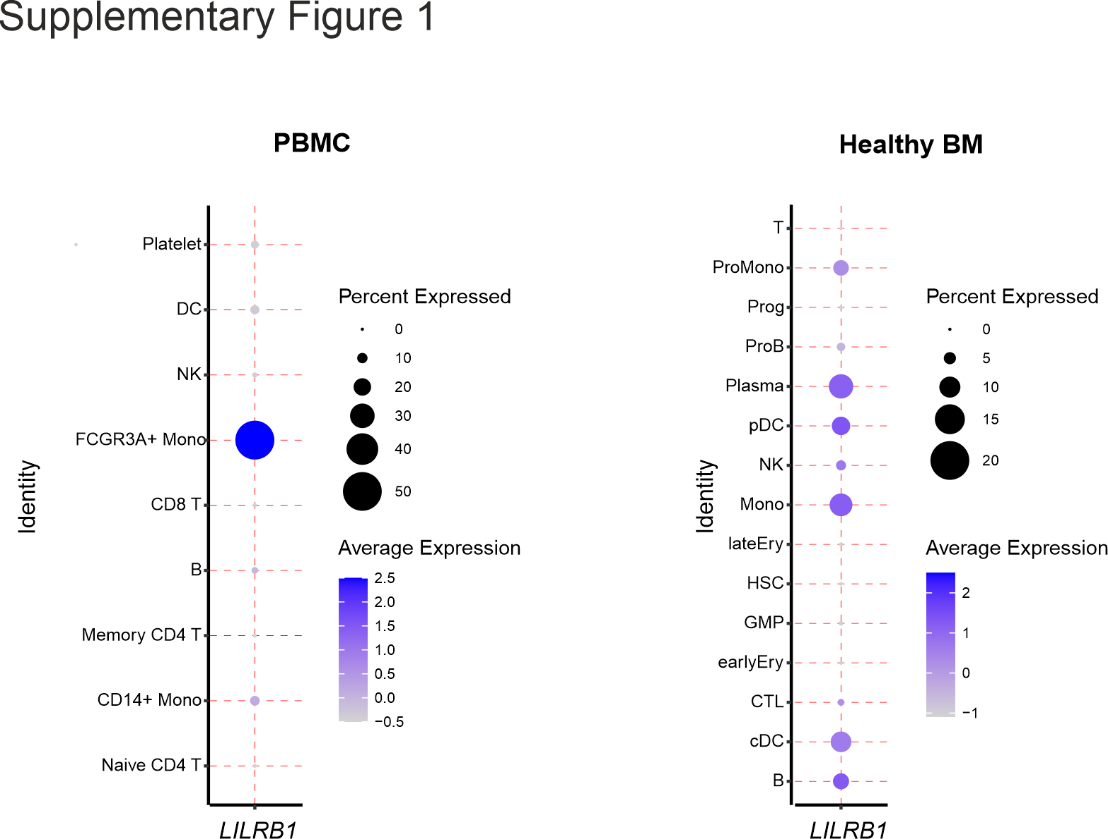


**Supplementary Figure 1.** ***LILRB1* mRNA expression in subpopulations of PBMC and BM cells.** *LILRB1* mRNA levels were assessed using the Tabula Sapiens database, containing single-cell transcriptomic data from healthy donors, across PBMC population (left panel) and bone marrow cell (BM) population (right panel). Dot size represents the percentage of cells expressing *LILRB1*, while blue color intensity indicates expression levels.


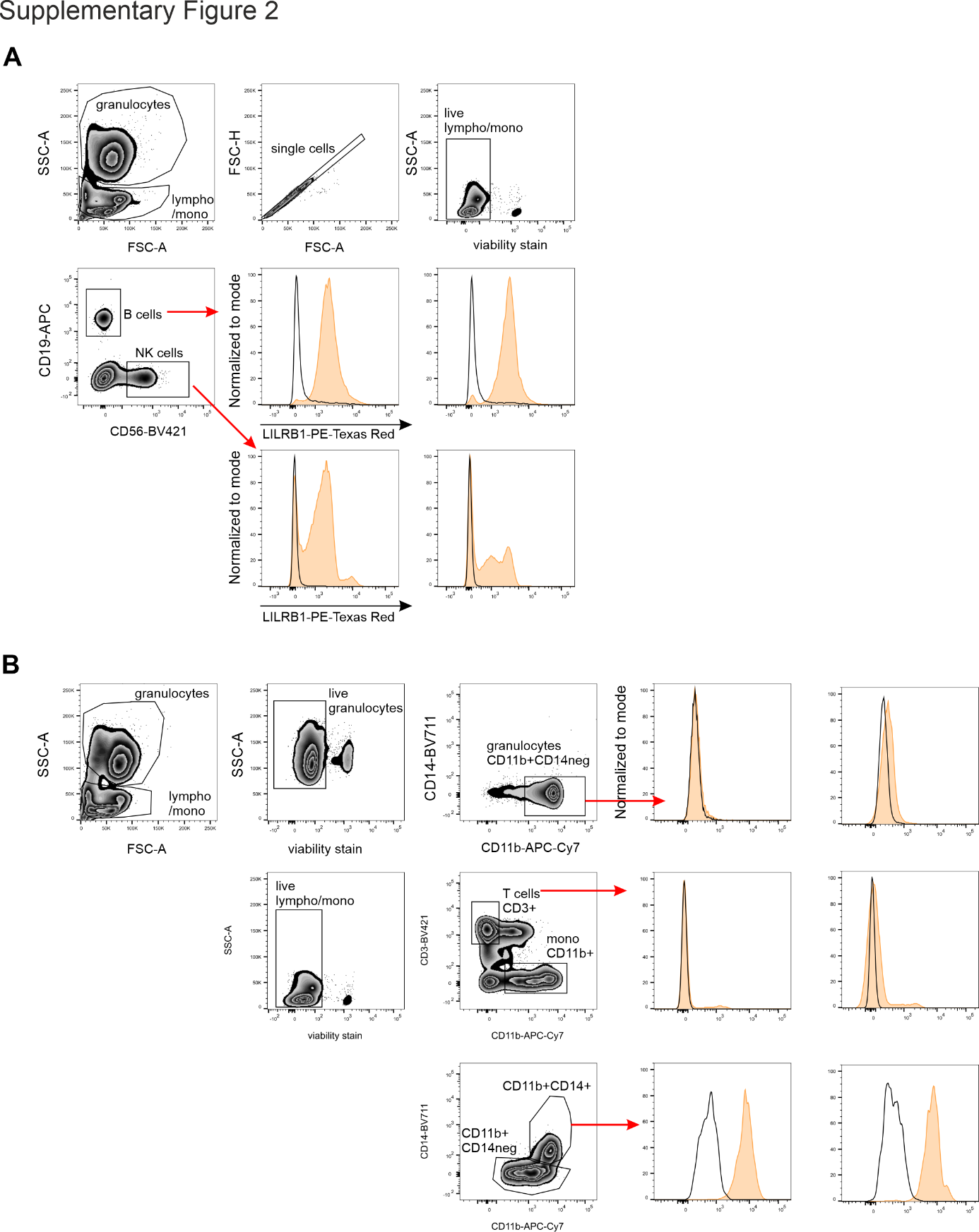


**Supplementary Figure 2.** **Gating strategy for flow cytometry analysis of LILRB1 expression on various peripheral blood leukocytes.** Following red cell lysis, peripheral blood from healthy donors (n=2) was stained first with a viability dye and then with anti-CD19, anti-CD56, anti-CD3, anti-CD14 and anti-CD11b antibodies. Sequential gating for B cells (CD19^+^ cells) and NK cells (CD56^+^ cells) identification **(A)**, and granulocytes (CD11b^+^CD14^-^), T cells (CD3^+^ cells) and monocytes (CD11b^+^CD14^+^ cells) identification **(B)** is shown on the representative graphs.


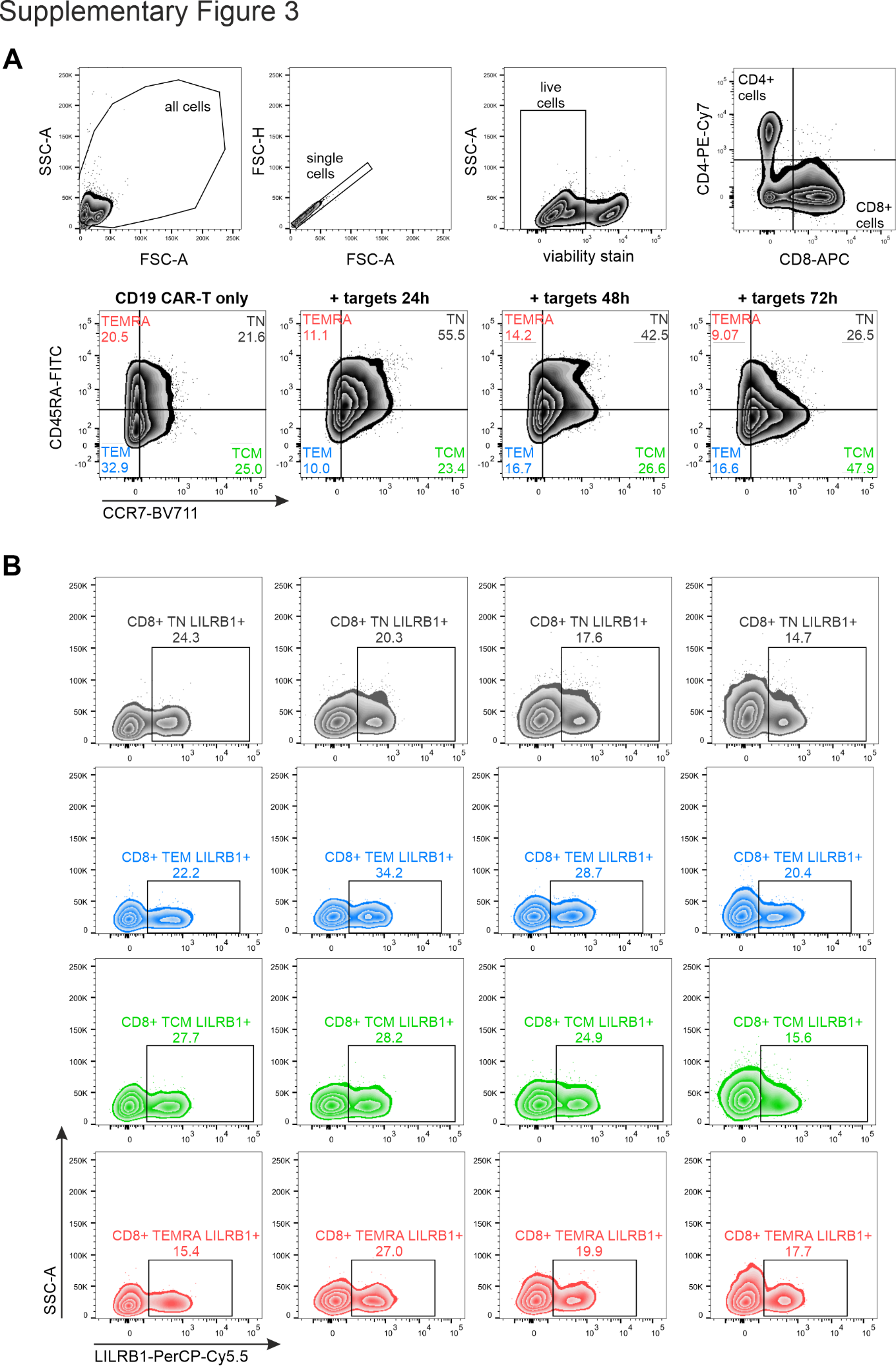


**Supplementary Figure 3. Evaluation of LILRB1 expression on antigen-activated CAR-T cells.** **A.** Representative flow cytometry gating strategy for the detection of LILRB1 levels in various subsets of CD19 CAR-T cells cultured alone (CD19 CAR-T only) or exposed to CD19^+^ Raji cells (+ targets) for 24, 48 and 72 hours at 1:1 E:T ratio. The subsets of T cells were determined based on the CD45RA and CCR7 levels and classified as TEMRA (terminally differentiated T cells), TN (naïve T cells), TEM (effector memory T cells), and TCM (central memory T cells). For each sample, the gating was adjusted to CD45RA and CCR7 fluorescence minus one (FMO) control. The experiment was performed using CAR-T cells from 2 donors (n=2). **B.** Representative dot plots showing the percentage of LILRB1^+^ cells among various subsets of CD8^+^ CD19 CAR-T cells unexposed or exposed to target cells for the indicated time.


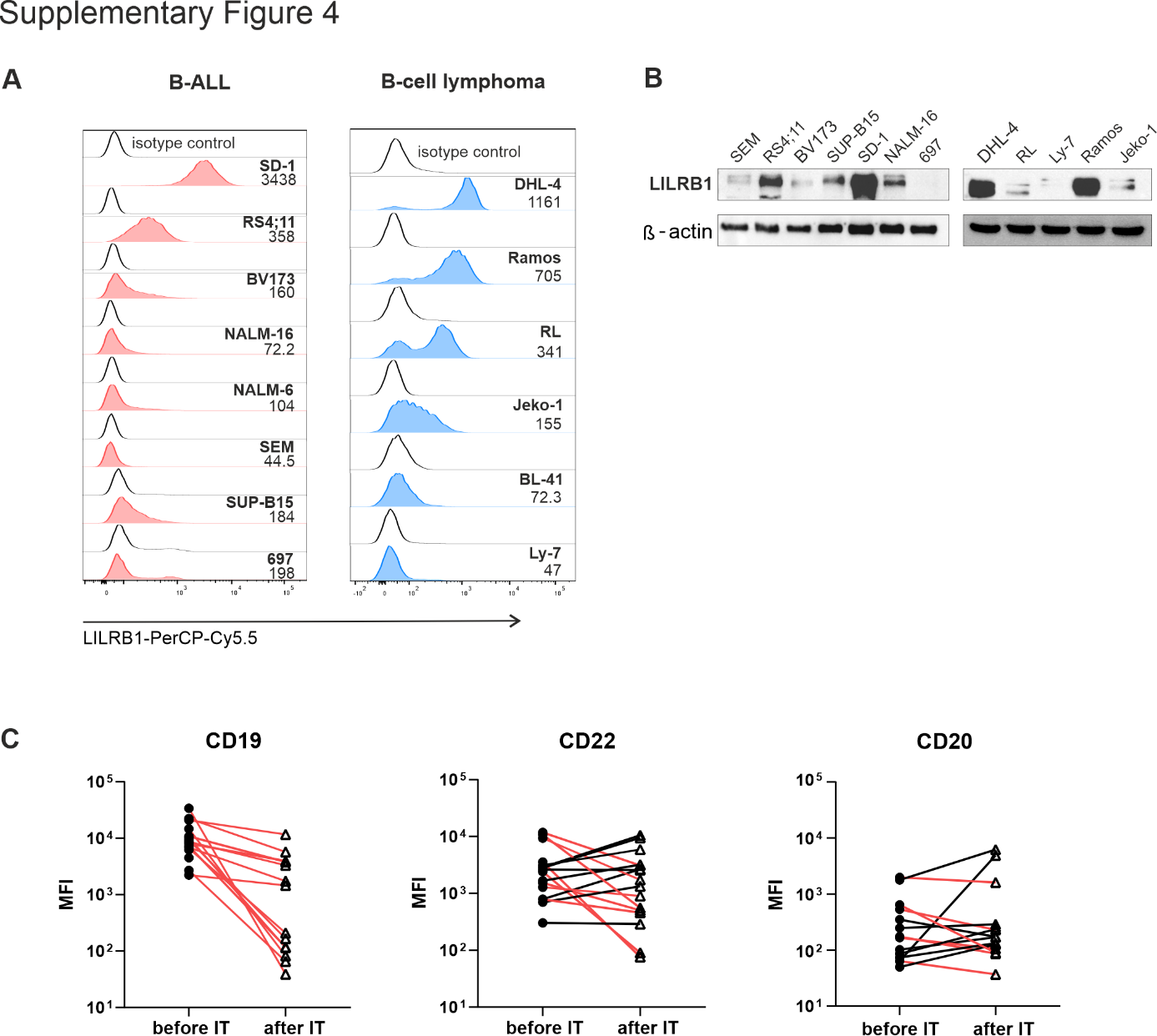


**Supplementary Figure 4.** **LILRB1 expression in B-ALL and B-NHL cell lines and the expression of CD19, CD22 and CD20 after CD19-targeted immunotherapy.** **A.** LILRB1 expression was evaluated by flow cytometry on eight B-ALL cell lines (left panel) and six B-NHL cell lines (right panel). The cells were stained with clone HP-F1 of anti-LILRB1 Ab. Mean fluorescence intensity (MFI) for each sample is shown next to the corresponding histogram. **B.** LILRB1 expression was evaluated by Western Blotting analysis in seven B-ALL cell lines (left panel) and five B-NHL cell lines (right panel). β-actin was used as a loading control. **C.** Levels of CD19, CD22, and CD20 were assessed in B-ALL cells before and after CD19-targeted immunotherapy (blinatumomab or CD19 CAR-T cells). The red coloring of the lines indicates a decrease in antigen expression.


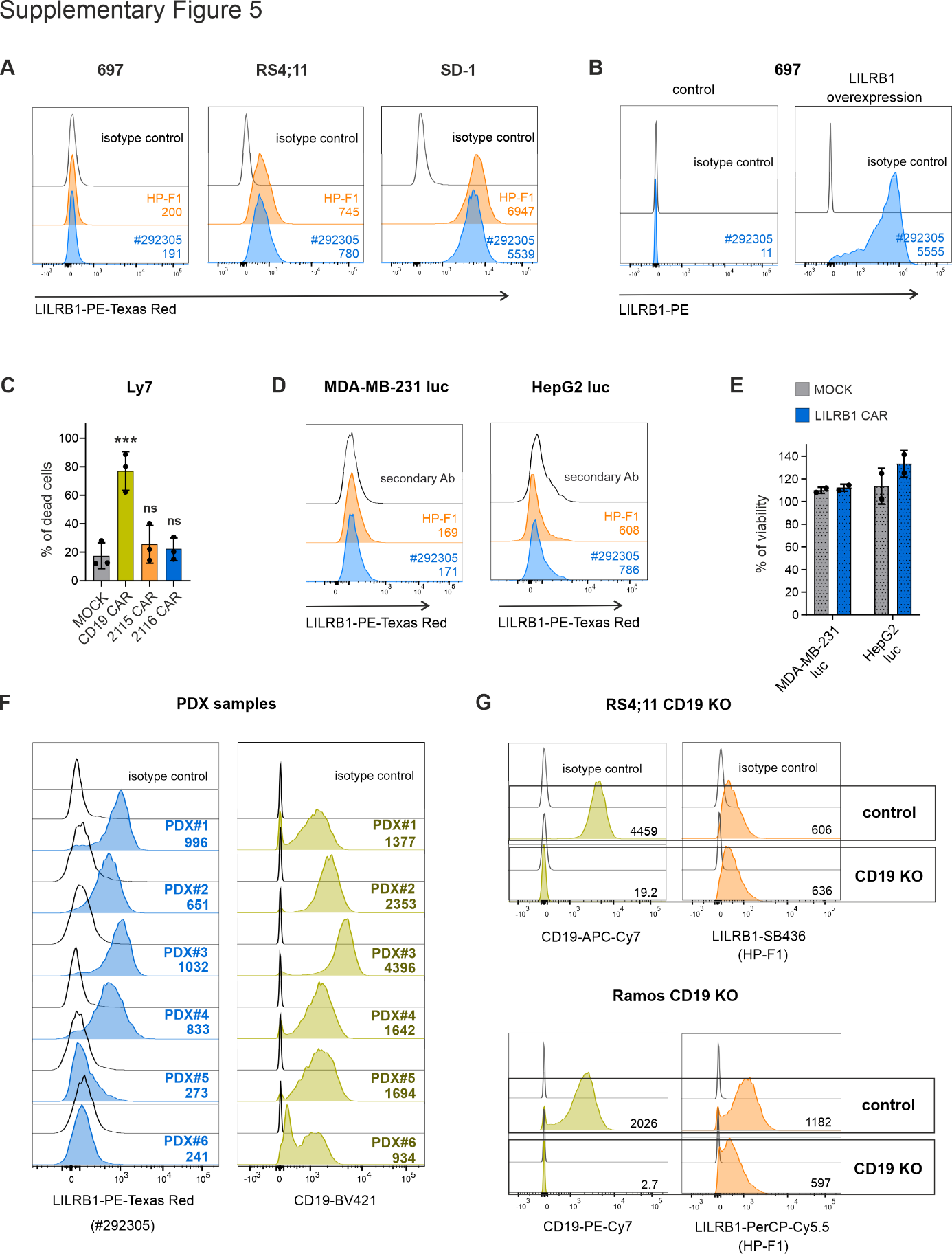


**Supplementary Figure 5.** **Verification of LILRB1 CAR-T cell specificity using target cells with varying levels of LILRB1 expression.** **A.** Comparison of LILRB1 surface levels using two different clones of anti-LILRB1 mAbs. Cell lines with low (697), moderate (RS4;11), and high (SD-1) levels of LILRB1 expression were used to compare selected clones of mouse anti-human LILRB1 monoclonal antibodies. The cells were stained with unconjugated clone HP-F1 or clone #292305 of anti-LILRB1 Ab and next with secondary donkey anti-mouse Ab conjugated with fluorochrome. LILRB1 expression was then evaluated by flow cytometry. Mean fluorescence intensity (MFI) for each sample is shown next to the corresponding histogram. **B.** *LILRB1* gene overexpression in 697 cells was generated by retroviral transduction and the results were validated by flow cytometry. MFI for each sample is shown next to the corresponding histogram. **C.** Cytotoxicity of LILRB1 CAR-T cells against LILRB1^low/-^ B-NHL cell line, Ly7, was assessed by flow cytometry-based killing assay. CAR-T cells and CTV-labeled target cells were co-cultured for 24 hours at 1:1 effector:target (E:T) ratio. The samples were then stained with propidium iodide (PI) and the percentage of dead CTV^+^PI^+^ target cells were determined. Data shows mean ± SD from n = 3 donors, *P* values were calculated using ordinary one-way ANOVA with Dunnett’s multiple comparisons test (MOCK vs. CAR-T). **D.** LILRB1 expression in breast cancer cell line (MDA-MB-231) and hepatoma cell line (HepG2) was evaluated by flow cytometry. The cells were stained either with HP-F1 clone or #292305 clone of anti-LILRB1 Ab. MFI for each sample is shown next to the corresponding histogram. **E.** Cytotoxicity of LILRB1 CAR-T cells against breast (MDA-MB-231) and liver (HepG2) cancer cell lines was assessed using a luciferase-based killing assay. CAR-T cells and luciferase-expressing target cells were co-cultured for 24 hours at 2:1 E:T ratio. The percentage of viable target cells was determined by measuring luminescence signal. Data shows mean ± SD from n = 2 donors. **F.** LILRB1 and CD19 expression on the B-ALL PDX cells used for the mass spectrometry (MS) analysis was evaluated by flow cytometry using clone #292305 of anti-LILRB1 Ab. MFI for each sample is shown next to the corresponding histogram. **G.** CD19 knockout (KO) in RS4;11 and Ramos cells was generated using the CRISPR-Cas9 technology. The levels of CD19 and LILRB1 expression were evaluated by flow cytometry in control (sgNTC) and KO cells.


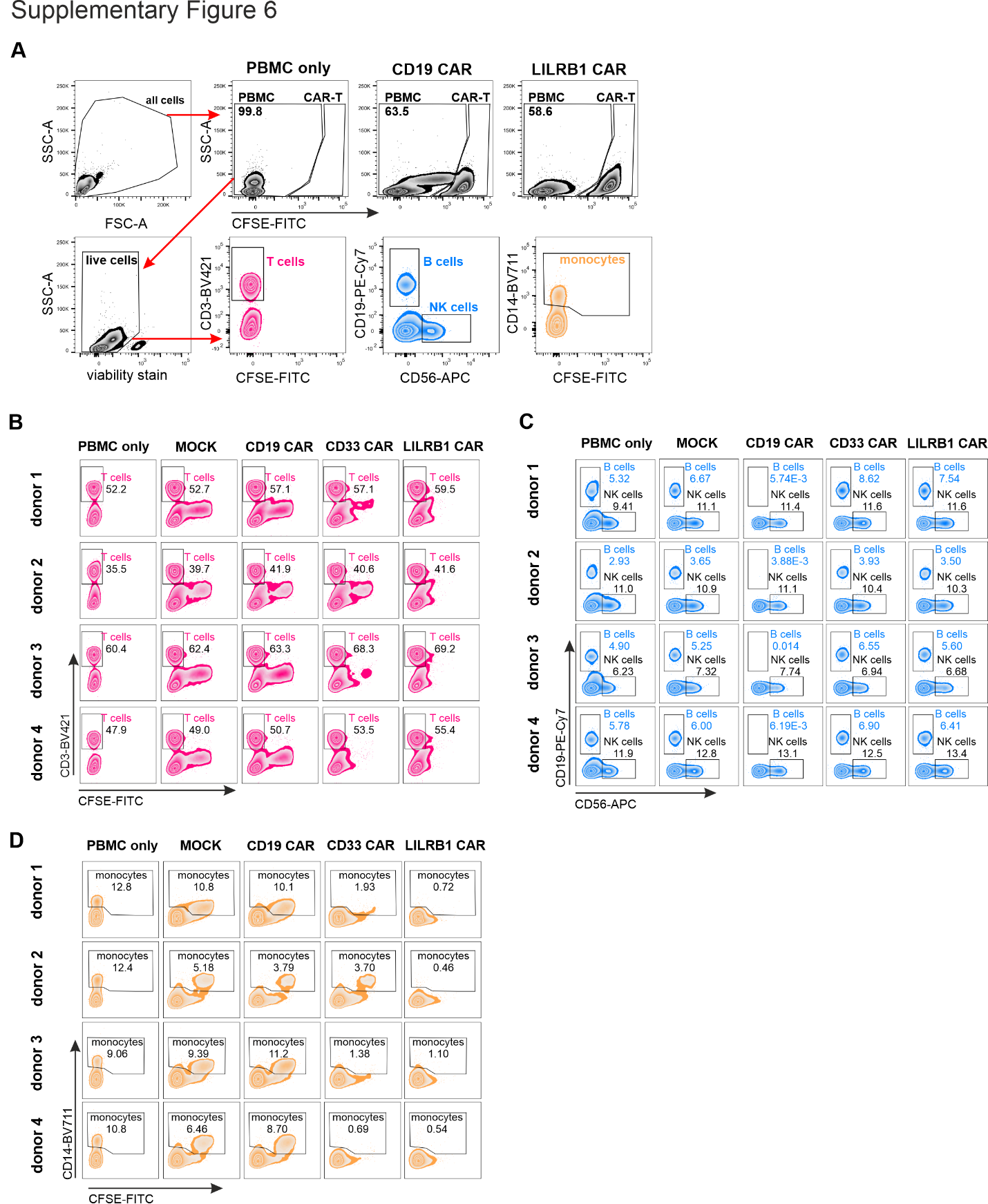
**Supplementary Figure 6. Evaluation of LILRB1 CAR-T cell cytotoxicity against PBMC subpopulations.** **A.** Representative gating strategy from CAR-T cytotoxicity assay against PBMC isolated from healthy donors (n=4). CFSE-labeled not transduced (MOCK) T cells, CD19, CD33, and LILRB1 CAR-T cells were incubated with PBMC at the 1:2 E:T ratio for 24 hours. Specific cell populations were determined by flow cytometry using anti-CD3 antibody (T cells), anti-CD19 antibody (B cells), anti-CD56 (NK cells), and anti-CD14 antibody (monocytes). Dot plots depict the percentage of T cells **(B)**, B cells/NK cells **(C),** and monocytes **(D)** present in each PBMC sample after exposure to MOCK T cells, CD19, CD33, and LILRB1 CAR-T cells.


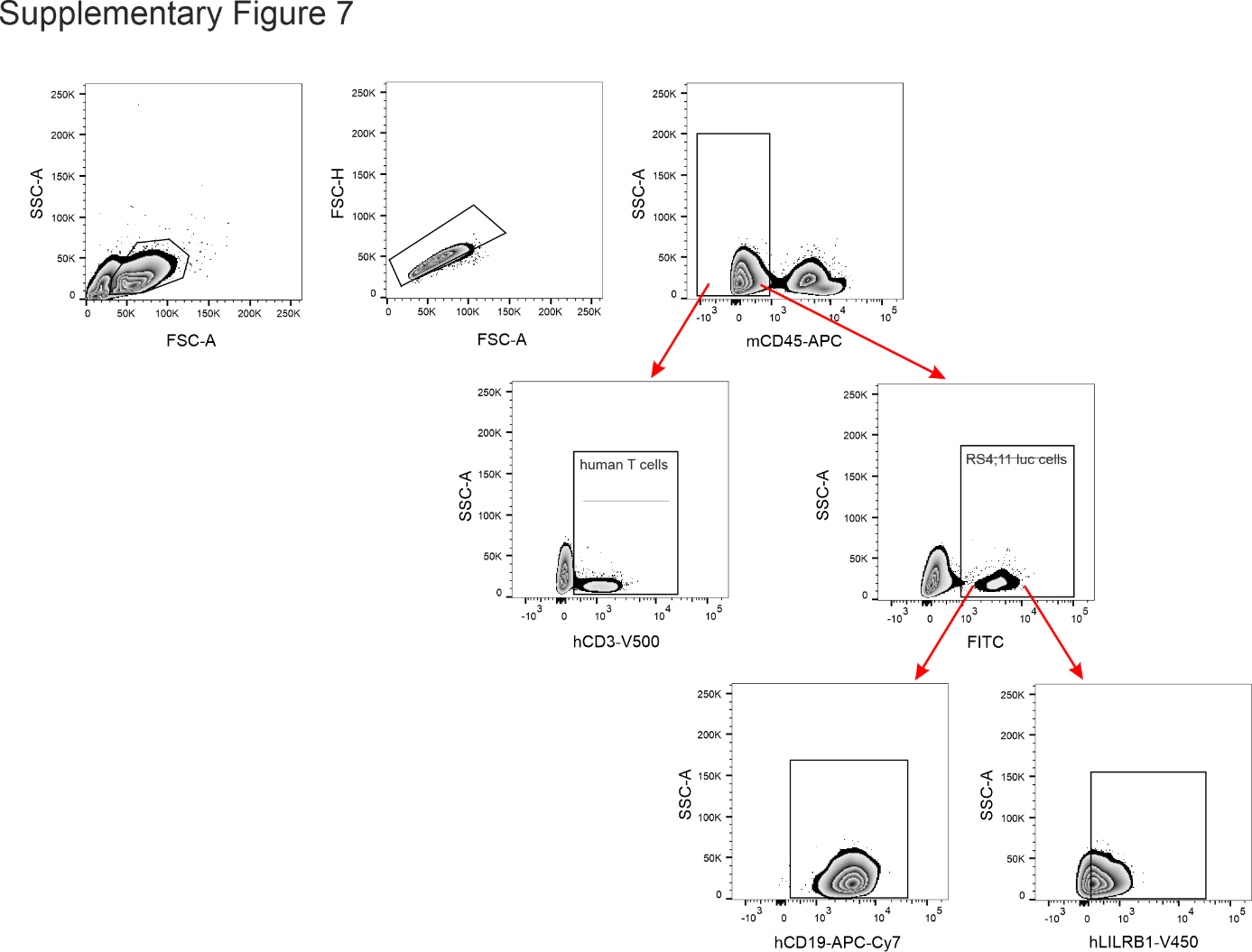


**Supplementary Figure 7. Gating strategy for flow cytometry analysis of LILRB1 CAR-T cells *in vivo* efficacy against B-ALL cells.** Gating strategy for RS4;11 GFP^+^ cells and human T cells identification in the samples obtained from the spleens and bone marrow of mice treated with LILRB1 or CD19 CAR-T cells. The mice were sacrificed when they reached the humane endpoint criteria. The spleens and bone marrow were isolated and homogenized. Following red cell lysis, the cells were stained with anti-mouse CD45, anti-human CD3, anti-human LILRB1, and anti-human CD19 antibodies.


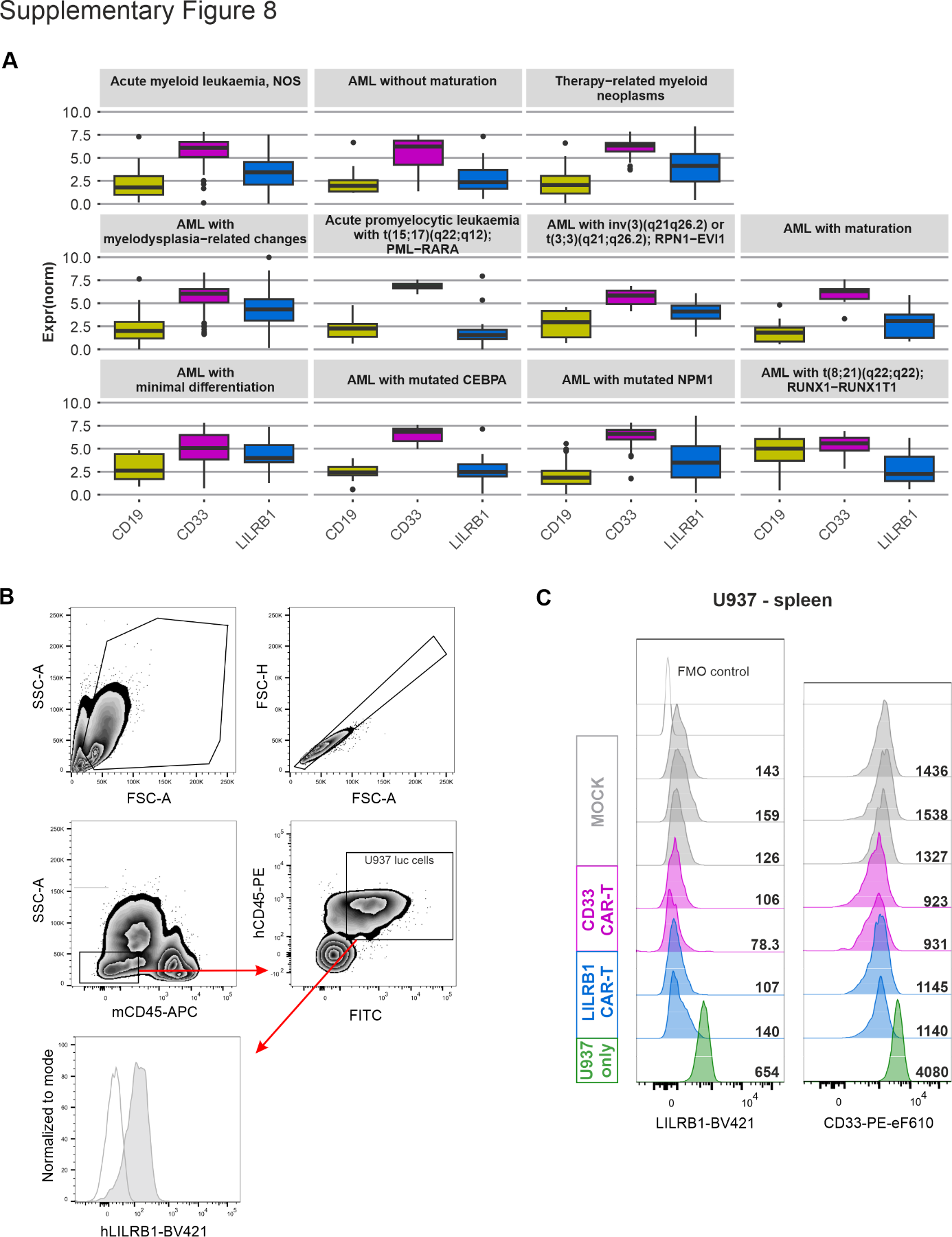


**Supplementary Figure 8.** **LILRB1 expression in AML patients and the evaluation of LILRB1 CAR-T cells’ potential to eliminate AML cells.** **A.** *LILRB1* mRNA expression in various subtypes of AML was compared to the expression of CD19 (negative control) and CD33 (positive control) expression. **B.** Gating strategy for U937 GFP^+^ cells identification in the samples obtained from the spleens of mice treated with LILRB1 CAR-T cells or CD33 CAR-T cells. The mice were sacrificed when they reached the humane endpoint criteria. The spleens were isolated and homogenized. Following red cell lysis, the cells were stained with anti-mouse CD45, anti-human LILRB1, and anti-human CD33 antibodies. **C.** LILRB1 expression (left panel) and CD33 expression (right panel) on U937 cells residing in the spleens of the mice following anti-LILRB1 or anti-CD33 CAR-T treatment was evaluated by flow cytometry. MFI for each sample is shown next to the corresponding histogram.

**Supplementary Materials and Methods**

**HUMAN CELL LINES**

The human B-ALL (SD-1, RS4;11, BV-173, NALM-16, SEM, SUP-B15, 697), B-NHL (DHL-4, Ramos, RL, Jeko-1, BL-41, Ly-7), and AML (U937, MV4;11, OCI-AML3, THP-1, Kasumi-1, HL-60) cell lines were obtained either from DSMZ or ATCC collections. B-ALL and B-NHL cells were maintained in RPMI-1640 (Gibco) supplemented with 10% fetal bovine serum (FBS; HyClone) and 1% penicillin/streptomycin (pen/strep) (complete RPMI medium). MV4;11 cells were cultured in Modified Dulbecco’s Medium + 20% FBS, OCI-AML3 in alpha-MEM + 20% FBS, and HL-60 in the Iscove's Modified Dulbecco's Medium + 20% FBS (all supplemented with pen/strep). The remaining AML cell lines were maintained in complete RPMI medium. All cells were cultured in a humidified atmosphere at 37°C, 5% CO_2_. The cells were routinely checked for Mycoplasma contamination.

**GENERATION OF B-ALL PDX FOR CELL SURFACE PROTEIN LABELING**

The patient samples were used following the patients’ written consent and approval of Bioethics Committee of Medical University of Warsaw (KB/44/2015). Approval for the animal experiments was given by the Ethics Committee of the Medical University of Warsaw (095/2019). Cryopreserved KMT2Ar BCP-ALL PDX were thawed and injected intravenously to 4-8 weeks old NOD.Cg-*Prkdc^scid^ Il2rg^tm1Wjl^*/SzJ (NSG) mice (Charles River Laboratories) via tail vein injections (2.0 –5.0 × 10^6^ cells/mouse) as described previously ^1,2^. The whole blood staining with anti‐mCD45 (clone 30-F11, eBioscience, Thermo Fisher Scientific, cat. no. 17-0451-82), anti‐hCD45 (clone HI30, eBioscience, Thermo Fisher Scientific, cat. no. 12-0459-42), anti‐hCD19 (clone HIB19, eBioscience, Thermo Fisher Scientific, cat. no. 11-0199-42) antibodies was performed to evaluate the engraftment of leukemic cells by flow cytometry. The mice were humanely killed when the percentage of human blasts in peripheral blood reached around 100% when compared to mouse CD45^+^ cells. B-ALL PDX cells were isolated from the spleens of leukemic mice and subjected to further processing *ex vivo*.

**SAMPLE PREPARATION FOR LC-MS ANALYSIS**

NeutrAvidin Agarose resin was suspended in 50 µL of ABC buffer. Before on-bead digestion, cysteines were reduced by 1 h incubation with 5 mM tris(2-carboxyethyl)phosphine (TCEP) at 60°C followed by 15 min incubation at a room temperature with 20 mM methyl methanethiosulfonate (MMTS). On-bead digestion was performed overnight at 37°C with 1 µg of trypsin (Promega, cat. no. V5111). Peptide solutions were transferred to the new tubes and the resin was further rinsed with 50 µl of ABC buffer. Pulled eluates were dried in SpeedVac and reconstituted in 20 µl of 0.1% formic acid (FA) and 2% acetonitrile (ACN) in water by 15 min sonication. Peptides were cleaned from the detergents using a modified single-pot solid-phase-enhanced sample preparation (SP3) protocol [10.1038/s41596-018-0082-x]. SP3 bead mix was prepared from equal parts of Sera-Mag Carboxyl hydrophilic and hydrophobic particles (GE Healthcare, cat. no. 09-981-121 and 09-981-123) in a working concentration of 5 µg/µl each. 24 µl of SP3 mix was then added to the samples along with 840 µl of ACN. Bound peptides were washed three times on a magnet with ACN and eluted from the beads with 2% ACN in MS-grade water. Peptides were dried in SpeedVac and resuspended in 50 µl of 0.1% TFA in water.

**SAMPLE ANALYSIS BY LC-MS**

Samples were analyzed using LC-MS system composed of Evosep One (Evosep Biosystems) coupled to an Orbitrap Exploris 480 mass spectrometer (Thermo Fisher Scientific). 20 µl of each sample was loaded onto Evotips C18 trap columns (Evosep Biosystems) according to the manufacturer’s protocol. Chromatography was performed using the 88 min (15 samples per day) method on the EV1106 analytical column (Dr Maisch C18 AQ, 1.9 µm beads, 150 µm ID, 15 cm long, Evosep Biosystems) with a flow rate of 220 nl/min. Data was acquired in a positive mode data-dependent manner and up to 40 precursors per cycle were chosen for fragmentation. MS1 resolution was set at 60 000 with a normalized AGC target of 300%, an Auto maximum inject time, and a scan range of 300 to 1600 m/z. For MS2, a resolution was set at 15 000 with a Standard normalized AGC target and an Auto maximum inject time. Dynamic exclusion was set at 20 s with an allowed mass tolerance of ±10 ppm and the precursor intensity threshold at 5e3. Precursors within 1.6 m/z window were fragmented in HCD mode with a normalized collision energy of 30%. The spray voltage was set to 2.1 kV, the funnel RF level at 40, and the heated capillary temperature at 275 °C.

**LC-MS DATA PROCESSING PROTOCOL**

Raw MS files were processed with MaxQuant (version 2.1.1) using canonical *Homo sapiens* database derived from Swissprot (version 2021_01, 20396 sequences) with fixed modification – Methylthio (C) and variable modification – Oxidation (M), enzyme – Trypsin and 2 missed cleavages. LFQ quantification was performed with classic normalization.

Further analysis was performed in Perseus (version 1.6.15). Potential contaminants, proteins from the reverse database and identified only by site were removed. At least 2 valid values in at least one group were required to a protein to be considered identified and pass for further analysis stages. Proteins described in Gene Ontology: Cellular Component with selected group of membrane-related terms (GO:0031012 “extracellular matrix”, GO:0005886 ”plasma membrane”, GO:0005576 “extracellular region”, GO:0005615 “extracellular space”, GO:0009986 “cell surface”, GO:0070062 “extracellular exosome”) were marked in categorical column (is_membrane). For each PDX, missing value replacement from normal distribution and t-test with permutation-based FDR was performed to find proteins significantly enriched relative to controls (non-biotinylated counterparts), with q-value threshold of 0.05. Proteins absent in non-biotinylated controls but present in at least 2 out of 4 biotinylated replicates were treated separately in categorical terms. For the following protein selection, the list of proteins identified in biotinylated samples only and the list of proteins observed to be significantly enriched in biotinylated samples compared to non-biotinylated controls were combined. From this group, 1409 proteins were selected as membrane-associated according to the UniProt database. Next, 945 membrane-associated proteins were identified as present in a minimum of 3 out of 6 analyzed PDX.

This subset of 945 proteins was subsequently analyzed in the context of tissue specificity using TissueEnrich R package (https://pubmed.ncbi.nlm.nih.gov/30346488/) with Human Protein Atlas (HPA) and GTEx databases used as crucial references. A total of 93 proteins were discerned as being expressed in lymphoid tissues, falling under the categories of Tissue-Enriched, Tissue-Enhanced, and Group-Enriched. Following a thorough manual curation in HPA and an extensive review of relevant research literature, the final list was refined to identify 18 lymphoid tissue-specific proteins, thus representing potential targets for CAR therapies.

**FLOW CYTOMETRY ANALYSIS OF CELL LINES, PRIMARY CELLS AND PDX**

***Detection of surface protein levels in cell lines and B-ALL PDX samples***

For cell surface markers detection, 0.5 × 10^6^ cells were stained with BD Horizon™ Fixable Viability Stain 510 (BD Biosciences, cat. no. 564406) for 15 minutes in PBS, washed with EasySep buffer (PBS with 2% FBS and 1mM EDTA) and incubated with BD Pharmingen™ Human BD Fc Block™ (BD Biosciences, cat. no. 564220) for another 15 minutes. Next, the cells were stained with either fluorochrome-conjugated or unconjugated anti-human LILRB1 antibodies (HP-F1 or #292305 clones; cat. numbers: 46-5129-42 - Thermo Fisher Scientific, FAB20171P-100 - Bio-techne R&D, 16-5129-82 - Thermo Fisher Scientific, MAB20171 - Bio-techne R&D). Fluorochrome-conjugated antibodies were added in 50 µl total volume of PBS, incubated for 25 minutes at room temperature and then washed in PBS. Alternatively, cells were incubated with unconjugated antibodies for 30 minutes on ice, washed twice with PBS, and then incubated with donkey anti-mouse IgG (H+L) secondary antibody (Thermo Fisher Scientific, cat. no. #A10037) for 20 minutes on ice. Following the final wash in PBS, the samples were analysed using BD LSRFortessa™ Cell Analyzer (BD Biosciences).

***Detection of LILRB1 levels in blood of healthy donors***

Peripheral blood of healthy donors was obtained from the Regional Blood and Hemotherapy Center in Warsaw. From each donor, 5 ml of whole blood was added to a falcon tube containing 40 ml of Gibco™ ACK Lysing Buffer (Thermo Fisher Scientific, cat. no. A1049201) and incubated for 5 minutes at room temperature. Next, the samples were centrifuged at 300 × g for 5 min and washed with 20 ml of cold PBS. The lysis was repeated and followed by three washing steps in PBS. Either 2.0 × 10^6^ (for T cells and monocytes detection) or 4.0 × 10^6^ cells (for B cells and NK cells detection) were then transferred to FACS tubes and stained with BD Horizon™ Fixable Viability Stain 510 (BD Biosciences, cat. no. 564406) for 15 minutes in PBS, washed with EasySep buffer (PBS with 2% FBS and 1mM EDTA) and incubated with BD Pharmingen™ Human BD Fc Block™ (BD Biosciences, cat. no. 564220) for 15 minutes. Next, anti-LILRB1 functional grade antibody (HP-F1 clone; eBioscience, Thermo Fisher Scientific, cat. no. 16-5129-82) was added to the samples and they were incubated for 30 minutes on ice. The cells were then washed twice in PBS and incubated with donkey anti-mouse IgG (H+L) secondary antibody (Thermo Fisher Scientific, cat. no. A10037) for 20 minutes on ice. Following that time, the cells were washed in PBS and stained with following fluorochrome-conjugated antibodies: anti-CD19 (BD Biosciences, cat. no. 555415), -CD56 (BioLegend, cat. no. 318328), -CD3 (BD Biosciences, cat. no. 562427), -CD14 (eBioscience, Thermo Fisher Scientific, cat. no. 67-0149-42), and CD11b (BD Biosciences, cat. no. 557754). Finally, the samples were washed in PBS and subjected to FACS analysis with BD LSRFortessa™ Cell Analyzer (BD Biosciences).

***Detection of LILRB1 levels in PBMC-derived macrophages***

Peripheral blood mononuclear cells (PBMCs) were isolated from buffy coats of healthy donors (obtained from the Regional Blood and Hemotherapy Center in Warsaw) through density-gradient centrifugation following a standard protocol. Next, CD14^+^ monocytes were isolated using CliniMACS CD14 microbeads, and LS separation magnetic columns (MiltenyiBiotec, cat. no. 130-019-101). 6.0 × 10^6^ of isolated monocytes were then seeded onto non-adherent dish culture in full RPMI-1640 medium supplemented with 20% FBS, antibiotics (100 U/mL penicillin, 100 μg/mL streptomycin), and 25 ng/ml human macrophage colony-stimulating factor (M-CSF, Immunotools, cat. no. 11343117). The cells were maintained in a humidified atmosphere at 37ᵒC and 5% CO_2_. Following 3 days, the new portion of full RPMI medium with 25 ng/ml M-CSF was added. After an additional 4 days, the cells were detached using Cellstripper™ (Corning, cat. no. 25-056-CI) and stained as described in the “Detection of surface protein levels in cell lines and B-ALL PDX samples”.

***Detection of LILRB1 levels in normal/regenerating bone marrow***

Normal/regenerating bone marrow (BM) samples from adult patients with T cell acute lymphoblastic leukemia or multiple myeloma in confirmed remission, 1-2 years after allo- and autologous hematopoietic stem cell transplantation were collected at the Institute of Hematology and Transfusion Medicine in Warsaw. BM samples were incubated with a mixture of fluorescencently‐labeled monoclonal antibodies: anti-CD38 (BD Biosciences, cat. no. 340909), -CD34 (BD Biosciences, cat. no. 345802), -CD33 (BD Biosciences, cat. no. 333146), -CD19 (BD Biosciences, cat. no. 341113), -CD45 (BD Biosciences, cat. no. 642275), -LILRB1 (clone HP-F1, eBioscience, Thermo Fisher Scientific, cat. no. 17-5129-42) using a standard protocol. Briefly, after 15 minutes of incubation, erythrocytes were lysed with BD PharmLyse solution (BD Biosciences, cat. no. 555899) and then washed with PBS with 10% fetal bovine serum (BD Pharmingen™ Stain Buffer (FBS), BD Biosciences, cat. no. 554656). BD FACS Canto™ II System (BD Biosciences) was used for samples acquisition and analysis of data files was conducted with FlowJo™ Software v.10.7.1 (BD Life Sciences). Singlets were selected on FSC-H/FSC-A and mononuclear cells were selected on FSC/SSC. After CD33^+^ and CD19^+^ cells exclusion, a population of CD34^+^ and CD45^+^ cells was gated. Hematopoietic stem and progenitor cells (HSPC) were further identified based on CD38 expression. The level of LILRB1 expression was quantified by median fluorescence intensity (MFI).

***Detection of LILRB1 levels in HSPC mobilized to peripheral blood***

For LILRB1 detection in HSPC, mobilized peripheral blood samples from healthy individuals were used. The samples were collected at the Hospital Clinic of Barcelona following standard protocol after an inform consent was signed (Institutional Review Board approval no HCB/2019/1032). Apheresis collection bags with cryopreserved samples were thawed in a water bath and the samples were centrifuged at 250 × g for 5 minutes. The supernatants were discarded, and the cells were washed twice in PBS. Next, the cells were incubated with DNase I Solution (Roche, cat. no. 10104159001) at a concentration of 100 µg/mL at room temperature for 15 minutes and washed twice with PBS with addition of 2% FBS. Aggregated suspensions were filtered through a 40 µm cell strainer for optimal results. The cell number and viability were determined using Countess II Automated Cell Counter (Life Technologies). Next, cells were stained on ice with the following fluorochrome-conjugated antibodies: anti-CD34 (BD Biosciences, cat. no. 348811), -CD38 (BD Biosciences, cat. no. 555459), -CD33 (BD Biosciences, cat. no. 551378), -CD19 (BD Biosciences, cat. no. 562440), and -LILRB1 (Bio-techne R&D, cat. no. FAB20171P-100; clone 292305). Finally, the samples were washed once in PBS and subjected to analysis on a FACS Canto™ II cytometer (BD Biosciences). The gating strategy was the same as described above, for normal/regenerating BM samples.

***Detection of LILRB1 levels in activated primary T cells***

PBMCs were isolated from buffy coats of healthy donors through density-gradient centrifugation following a standard protocol. Next, primary T cells were isolated using EasySep™ Human T Cell Enrichment Kit (STEMCELL Technologies, cat. no. 19051) through negative selection. Isolated T cells were then stimulated with Dynabeads™ Human T-Activator CD3/CD28 for T Cell Expansion and Activation (Thermo Fisher Scientific, cat. no. 11131D) at 1:2 bead-to-cell ratio for 48 hours. The cells were maintained in complete RPMI medium with the addition of recombinant human IL-2 (Peprotech, cat. no. 200-02) at the final concentration of 100 U/ml. Following the indicated time, the unstimulated T cells (controls) and stimulated T cells were stained with Fixable Viability Stain 510 (BD Biosciences, cat. no. 564406) for 15 minutes and then washed once in the EasySep buffer (PBS with 2% FBS and 1mM EDTA). Next, the cells were incubated with BD Pharmingen™ Human BD Fc Block™ (BD Biosciences, cat. no. 564220) for 15 minutes and then stained for 25 minutes with the following antibodies: anti-LILRB1 (HP-F1 clone, eBioscience, Thermo Fisher Scientific, cat. no. 46-5129-42), -CD4 (BD Biosciences, cat. no. 557852), and -CD8 (BD Biosciences, cat. no. 564526). The samples were analyzed using BD LSRFortessa™ Cell Analyzer (BD Biosciences).

***Detection of LILRB1 levels in the subsets of CD19 CAR-T cells***

To determine the level of LILRB1 in CD19 CAR-T cells (pSEW-CARCD19-41BB; lentiviral system) upon target engagement, 4.0 × 10^6^ CAR-T cells were seeded onto 24-well plate and cultured alone, or co-cultured with CD19^+^ target cells (Raji) at 1:1 E:T ratio for 24, 48 or 72h. Following the indicated time, the cells were stained with Fixable Viability Stain 510 (BD Biosciences, cat. no. 564406) for 15 minutes and then washed once in the EasySep buffer (PBS with 2% FBS and 1mM EDTA). Next, the cells were incubated with BD Pharmingen™ Human BD Fc Block™ (BD Biosciences, cat. no. 564220) for 15 minutes and then stained for 25 minutes with the following antibodies: anti-LILRB1 (HP-F1 clone, eBioscience, Thermo Fisher Scientific, cat. no. 46-5129-42), anti-CD69 (BioLegend, cat. no. 310914), -CD4 (BD Biosciences, cat. no. 557852), -CD8 (BD Biosciences, cat. no. 561953), -CCR7 (BD Biosciences, cat. no. 566602), -CD45RA (eBioscience, Thermo Fisher Scientific, cat. no. 11-0458-42). Samples were then washed in PBS and the surface levels of selected proteins were evaluated using BD LSRFortessa™ Cell Analyzer (BD Biosciences). The gating strategy is presented on Supplementary Figure 3.

***Detection of LILRB1 levels in primary pediatric B-ALL samples***

The patient samples were used following the patients’ written consent and approval was given by the Ethical Committee at the Medical University of Silesia (PCN/0022/KB1/90/XV/20/21). LILRB1 expression was examined in 21 BCP-ALL patients at first diagnosis (male/female ratio 0.91) and in 7 BCP-ALL patients after relapse (male/female ratio of 0.4) without prior immunotherapy with blinatumomab or CD19 CAR-T cells and in one patient before and after CD19 CAR-T cell immunotherapy. Flow cytometric analyses were performed centrally at the Department of Pediatric Hematology and Oncology in Zabrze of the Medical University of Silesia in Katowice, including the first diagnosis, relapse, and relevant follow-up time points. EDTA-collected BM samples were prepared according to the standardized protocol developed by the EuroFlow Consortium (Euroflow Consortium. EuroFlow Standard Operating Procedure (SOP) for Sample preparation Version 1.7. https://euroflow.org/protocols/) and flow cytometer set up, calibration and data acquisition were concordant with the standardized procedure described previously ^3^. Briefly, BM samples were incubated with selected monoclonal antibodies (anti-CD19-PE-Cy7, clone J3.119, Beckman-Coulter, cat. no. IM3628; anti-CD45-V500, clone HI30, Becton Dickinson, cat. no. 560777; anti-CD34-PerCP-Cy5.5, clone 8G12, Becton Dickinson, cat. no. 347222; anti-CD22-PE, clone S-HCL-1, Becton Dickinson, cat. no. 333145; anti-CD10-APC, clone HI10a, Becton Dickinson, cat. no. 332777; anti-CD20-Pacific Blue, clone 2H7, Biolegend, cat. no. 302320; and anti-LILRB1 (CD85j, ILT2)-Super Bright 436, clone HP-F1, Thermo Fisher, cat. no. 63-5129-42) for 30 min at room temperature. Erythrocytes were lysed for 10 min with BD Lysing Solution (BD Biosciences, cat. no. 349202). After the lysis, the sample was washed with PBS solution supplemented with 10% fetal bovine serum and acquired with FACS Canto™ flow cytometer (BD Biosciences). For data analysis, Infinicyt software was used (BD Biosciences). In each data file, mean fluorescence intensity (MFI) values per marker were recorded for all cell populations identified in the sample, including leukemic blasts, together with their percentage distribution, after exclusion of cellular debris and cell doublets.

***Detection of LILRB1 levels in primary adult B-ALL, B-NHL, and M5 AML samples***

The patient samples were used following the patients’ written consent and approval was given by the Ethical Committee at the Institute of Hematology and Transfusion Medicine in Warsaw (41/2020, 42/2023). The study group consisted of 30 adult patients (male/female ratio of 1.2) diagnosed with B-ALL (n=12), ten patients with B-NHL i.e. marginal zone lymphoma (MZL) (n=6) and mantle cell lymphoma (MCL) (n=4), and with M5 acute myeloid leukemia (M-AML) (n=8). The diagnostic samples were collected at the Institute of Hematology and Transfusion Medicine in Warsaw. The median age at the day of diagnosis was 50, 74 and 70 years for B-ALL, B-NHL and M-AML, respectively. Anti-LILRB1 antibody (clone HP-F1, eBioscience, Thermo Fisher Scientific, cat. no. 17-5129-42) and APC-labeled isotypic control (clone P3.6.2.8.1, Invitrogen, cat. no. 17-4714-82) were incorporated into flow cytometry panels of antibodies routinely used in diagnosis of hematological malignancies in the Institute of Hematology and Transfusion Medicine in Warsaw. In the B-NHL panel, anti-LILRB1 antibody was included in 7-color test tube containing anti-CD20 (BD Biosciences, cat. no. 655872), anti-CD45 (BD Biosciences, cat. no. 655873), anti-CD43 (BD Biosciences, cat. no. 555475), anti-CD79b (Beckman Coulter, cat no.IM1612U), anti-CD5 (BD Biosciences, cat. No. 341109), anti-CD19 (BD Biosciences, cat. no. 341113) antibodies. In the B-ALL panel, anti-LILRB1 antibody was included in 6-color test tube containing anti-CD45 (BD Biosciences, cat. no. 655873), anti-CD58 (Beckman Coulter, cat no. IM1218U), anti-CD34 (BD Biosciences, cat. no. 347222), anti-CD19 (BD Biosciences, cat. no. 341113), anti-CD10 (BD Biosciences, cat. no. 655404) antibodies. In M-AML cases, 11-color tube containing anti-CD13 (BD Biosciences, cat. no. 562596), anti-CD45 (BD Biosciences, cat. no. 655873), anti-CD36 (Beckman Coulter, cat. no. B49201), anti-CD64 (Beckman Coulter, cat. no. IM3601U), anti-CD11c (BD Biosciences, cat. no. 658330), anti-CD117 (BD Biosciences, cat. no. 339217), anti-CD14 (BD Biosciences, cat. no. 333951), anti-HLA-DR (BD Biosciences, cat. no. 565127), anti-CD11b (BD Biosciences, cat. no. 742639), anti-CD15 (BD Biosciences, cat. no. 563838) and anti-LILRB1 antibodies were used. To perform cell surface staining, EDTA-collected BM samples were incubated with a mixture of labeled monoclonal antibodies using a standard protocol. Briefly, after 15 min incubation, erythrocytes were lysed with BD PharmLyse solution (BD Biosciences, cat. no. 555899) and then washed with PBS with 10% fetal bovine serum (BD Pharmingen™ Stain Buffer (FBS), BD Biosciences, cat. no. 554656). The cells were resuspended in PBS and acquired on the flow cytometer within 1 hour after preparation. BD FACS Canto™ II or BD FACS Lyric™ instruments (BD Biosciences) were used for samples acquisition. The instruments were set up according to EuroFlow Consortium standardized procedure ^3^ and calibrated daily using CS&T IVD Beads (BD Bioscience, San Jose, CA, USA) according to the manufacturer's instructions. At least 50 000 events were collected in each sample. After exclusion of cellular debris and cell doublets, pathological cells in B-NHL and B-ALL cases were determined by gating of CD19^+^ B-cells population and their characteristic abnormal immunophenotype. In M-AML cases first total monocytoid cells population with CD64 and CD36 expression was determined and pathological cells were further divided based on immunophenotype pattern of immature monocytoid cells: CD13^+dim^CD14^(-)^CD11c^+^HLA-DR^+^CD15^+^CD11b^+dim++^ and mature monocytoid cells: CD13^+^CD14^+^ CD11c^+^HLA-DR^+^CD15^(-)^CD11b^+^. The level of LILRB1 expression was quantified by median fluorescence intensity (MFI) and the percentage of neoplastic population exceeded an isotypic control for LILRB1.

***Detection of CD19, CD22, and CD20 levels in B-ALL patients before and after immunotherapy***

The analysis of the levels of CD19, CD22, and CD20 before and after CD19-directed immunotherapy, as presented in Supplementary Figure 4, was conducted retrospectively, using the flow cytometry data collected for diagnostic purposes. The study group consisted of 20 pediatric patients (male/female ratio of 1.22) primarily diagnosed with B-ALL. The median age on the day of B-ALL diagnosis was 3.9 years. Each of the 20 patients relapsed after completion of the frontline treatment which was followed by the introduction of immunotherapy (IT) with the use of blinatumomab or CAR-T therapy. B-ALL cells from BM samples collected at respective time points were stained with anti-CD19, anti-CD22, and anti-CD20 monoclonal antibodies.

**HUMAN T CELL ACTIVATION**

PBMCs were isolated from buffy coats of healthy donors through density-gradient centrifugation following a standard protocol. To activate human T cells, the total of 6 × 10^6^ isolated PBMCs were seeded onto 6-well plate per well, and incubated with 1µg/ml anti-CD3 (functional grade OKT3, eBioscience, Thermo Fisher Scientific, cat. no. 16-0037-85), and 1µg/ml anti-CD28 (functional grade CD28.6, eBioscience, Thermo Fisher Scientific, cat. no. 16-0289-85) antibodies at 37°C 5% CO_2_. Following 2-3 days, the cells were counted and prepared for further processing.

**TRANSDUCTION AND EXPANSION OF HUMAN T CELLS**

Until stated otherwise in particular methods descriptions, all experiments involving CAR-T cells were performed with effector cells produced using a retroviral platform for cell modification. Retroviral particles were produced in Hek-P cells as described previously ^4^. For the transduction, 0.3 × 10^6^ of activated T cells in 0.5 ml of complete RPMI medium were added to a 24-well plate coated with 50 µg/ml RetroNectin (Takara Bio, cat. no. T100B) and mixed with 0.5 ml of retroviral supernatant. The cells were spinoculated at 750 × g for 60 minutes at 32°C and then incubated at 37°C 5% CO_2_ for 24h. On the next day, the spinoculation was repeated using another 0.5 ml of retroviral supernatant. On the following day, the medium was replaced and Dynabeads™ Human T-Activator CD3/CD28 for T Cell Expansion and Activation (Thermo Fisher Scientific, cat. no. 11131D) was added to the T cells at 1:1 bead-to-cell ratio for 5-7 days. The modification efficiency was determined following 5-7 days post-transduction by evaluating the human CD34 (clone 4H11; eBioscience, Thermo Fisher Scientific, cat. no. 17-0349-42), or murine Fab surface expression (anti-F(ab')₂ fragment Alexa Fluor® 647, Jackson Immunoresearch, cat. no. 115-606-072).

**GENERATION OF LILRB1-OVEREXPRESSING 697 CELL LINE**

LILRB1 was amplified using a commercial vector (OHu23369D, Gene script) as a template and the following primers: CACCATGACCCCCATCCTCACGGTCC and TATCAATTGGTGGATGGCCAGAGTGGCGTAG to generate a compatible fragment for pENTR/TOPO (Invitrogen) where the STOP was replaced by a MunI site. After sequence checking, the modified insert was extracted and subcloned into a plasmid containing the sequence fused to truncated CD34 tag. This construct was finally recombined into a Gateway compatible pMP71 retroviral vector. 697 cells overexpressing LILRB1 were generated by retroviral transduction. The population of LILRB1^+^ cells was purified using FACS Aria III cell sorter (BD Biosciences).

**GENERATION OF CD19 KO CELL LINES**

Cell modification was performed using the CRISPR-Cas9 genome editing method and lentiviral system. The sgRNA sequence selected from the Brunello database was inserted by cloning into the pLentiCRISPR v2 lentiviral vector (Addgene #52961). For sgRNA-containing viral particles production, HEK293T cells were transfected with sgRNA lentiviral constructs encoding sgRNA (5’-ATGAAAAGCCAGATGGCCAG-3’) targeting exon 2 in the *CD19* gene, VSV-G envelope expressing plasmid pMD2.G (RRID: Addgene_12259), and lentiviral packaging plasmid psPAX2 (RRID: Addgene_12260). The 2.25:1 ratio of the FuGENE HD reagent to DNA was used for transfection. The experiments also included a control plasmid with a non-targeting sgRNA sequence (sgNTC, 5’- ACGGAGGCTAAGCGTCGCAA-3’). The cells were modified by double spinoculation with viral supernatants for 1 h (1250 × *g* at 32°C). After 3 days from transduction, the 5-day selection of modified cells was performed using puromycin (1.5 µg/ml – Ramos and 1 µg/ml – RS4;11). Afterward, to obtain a population of CD19-negative cells only, the modified cells were stained with anti-CD19 antibody (eBioscience, Thermo Fisher Scientific, cat. no. 25-0199-42 or eBioscience, Thermo Fisher Scientific, cat. no. 47-0199-42), and the negative population was sorted on BD FACS Aria III cell sorter (BD Biosciences).

**DEGRANULATION ASSAY**

Target cells were seeded onto a 96-well plate at density 0.2 × 10^6^ of cells per well. Effector cells were added to target cells at E:T ratio 1:2. A mix of anti-human CD107a antibody (BD Biosciences, cat. no. 555801), GolgiStop™ (BD Biosciences, cat. no. 554724) and GolgiPlug™ Protein Transport Inhibitor (BD Biosciences, cat. no. 555029) reagents was added to each well and the cultures were incubated overnight at 37°C 5% CO_2_. Upon incubation time, the cells were spun down, washed with PBS, and stained first with BD Horizon™ Fixable Viability Stain 510 (BD Biosciences, cat. no. 564406), and next for T cell markers: CD3 (clone UCHT1, BD Biosciences, cat. no. 562427) and CD8 (clone RPA-T8, BD Biosciences, cat. no. 555369). The degranulation of effector cells was then assessed using BD LSRFortessa™ Cell Analyzer (BD Biosciences).

**ANALYSIS OF IFN-γ AND TNF-α SECRETION**

Cytokine release by CAR-T cells was evaluated using ELISA assay. CAR-T cells and target cells were co-incubated for 24 h at 37°C 5% CO2 at 1:2 E:T ratio. The concentration of IFNγ and TNFα was then measured in the culture medium using Human IFN-γ ELISA Kit (Invitrogen, Thermo Fisher Scientific, cat. no. KHC4021) and Human TNF‑a ELISA Kit (Invitrogen, Thermo Fisher Scientific, cat. no. KHC3011) according to the manufacturers’ protocols. The samples were analyzed by PerkinElmer Multimode Plate Reader EnVision.

**FLOW CYTOMETRY-BASED KILLING ASSAY**

Target cells were stained with CellTrace™ Violet dye (Thermo Fisher Scientific, cat. no. C34557) according to manufacturer’s protocol and seeded onto 96-well plate at density 0.1 × 10^6^ of cells per well. Effector cells were added to target cells at various E:T ratios (0.5:1, 1:1, 2.5:1) and the cells were incubated for 24h at 37°C 5% CO_2_. To determine the percentage of dead target cells, propidium iodide (PI, Sigma-Aldrich, cat. no. P4864) was added upon incubation time at final concentration 1 µg/ml. Cytotoxicity of effector cells was evaluated using BD LSRFortessa™ Cell Analyzer (BD Biosciences).

**LUCIFERASE-BASED KILLING ASSAY**

Suspension target cell lines with luciferase expression were seeded onto a 96-well plate at density of 2.5 × 10^4^ cells per well. Adherent target cells were seeded at the same density onto 96-well black plates with a clear bottom (PerkinElmer, cat. no. 6005182). Effector cells were added to target cells at E:T ratio of 0.5:1 or 1:1, and the cells were incubated at 37°C 5% CO_2_. After 24h, 100 µl of suspension cultures was transferred onto white 96-well plate and 100 μl of the mix of Bright-Glo^TM^ Luciferase Assay (Promega, cat. no. E2620) was added. For the cultures with adherent target cells, 100 µl of medium was removed from black plates and replaced with 100 μl of the mix of Bright-Glo^TM^ Luciferase Assay. After 5 minutes of incubation, the bioluminescence signal was detected using Victor Plate Reader (PerkinElmer).

**CAR-T CYTOTOXICITY ASSAY AGAINST HEALTHY DONOR-DERIVED PBMC**

Effector untransduced (MOCK) T cells as well as CD19, CD33, and LILRB1 CAR-T cells were pre-labeled with CFSE CellTrace™ (Thermo Fisher Scientific, cat. no. C34554). Next, 1.0 × 10^6^ effector cells were plated onto a 24-well plate and co-cultured with PBMC (E:T - 1:2) isolated from 4 healthy donors. Following 24h, the cells were collected and washed twice in PBS. Then the cells were stained with Fixable Viability Stain 510 (BD Biosciences, cat. no. 564406) for 15 minutes and washed once in the EasySep buffer (PBS with 2% FBS and 1mM EDTA). Next, the cells were incubated on ice with BD Pharmingen™ Human BD Fc Block™ (BD Biosciences, cat. no. 564220) for 15 minutes and then stained for 25 minutes with the following mix of antibodies: anti-CD19 (eBioscience, Thermo Fisher Scientific, cat. no. 25-0199-42), -CD56 (BD Biosciences, cat. no. 555518), -CD3 (BD Biosciences, cat. no. 562427), -CD14 (eBioscience, Thermo Fisher Scientific, cat. no. 67-0149-42) for 30 minutes at room temperature. Samples were then washed in PBS and analyzed using BD LSRFortessa™ Cell Analyzer (BD Biosciences). The gating strategy is presented in Supplementary Figure 6.

**CFU ASSAY**

Effector T cells were co-cultured with non-autologous Bone Marrow Mononuclear Cells (BMNCs) for 6 hours in complete RPMI medium at an E:T ratio of 10:1. After culture, the cells were washed and resuspended in 400 µL of PBS 10% Human Serum Albumin (HSA). The solution was then mixed with 4 mL of complete methylcellulose media (MethoCult medium, STEMCELL Technologies, cat. no. Catalog # 04064), and 1.1 mL was transferred to each well of a 6-well plate (in triplicate). After 15 days, colony-forming unit–erythroid (CFU-E), burst-forming unit– erythroid (BFU-E), colony-forming unit–granulocyte, macrophage (CFU-GM), and colony-forming unit–granulocyte, erythrocyte, monocyte, megakaryocyte (CFU-GEMM) colonies were counted under a microscope.

**FLOW CYTOMETRY ANALYSIS OF MURINE TISSUES COLLECTED FROM *IN VIVO* STUDIES**

Spleens were smashed through the Falcon® 100 µm Cell Strainer (Corning, catalog no. 352360) using a syringe plunger. Bone marrow was extracted from femur and tibia of the mice by flushing it with PBS using a syringe with a needle. Isolated cells were washed with ice-cold PBS. Next, red blood cells were removed from the cell suspension using ACK Lysing Buffer (Thermo Fisher Scientific, catalog no. A1049201). The suspensions of single cells were then incubated with BD Pharmingen™ Human BD Fc Block™ (BD Biosciences, catalog no. 564220) for 15 minutes prior to the addition of antibodies. The cells from spleens and bone marrow of the mice injected with B-ALL cells (RS4;11) were stained for 25 minutes with anti-mouse CD45 (eBioscience, Thermo Fisher Scientific, catalog no. 17-0451-82), anti-human CD3 (BioLegend, catalog no. 300448), anti-human CD19 (eBioscience, Thermo Fisher Scientific, catalog no. 47-0199-42) and anti-human LILRB1 (HP-F1 clone, eBioscience, Thermo Fisher Scientific, catalog no. 62-5129-42). The samples were analysed using BD FACS Canto™ II Flow Cytometer (BD Life Sciences). The cells from spleens of the mice injected with AML cells (U937) were stained for 25 minutes with anti-mouse CD45 (eBioscience, Thermo Fisher Scientific, catalog no. 17-0451-82), anti-human CD45 (eBioscience, Thermo Fisher Scientific, catalog no. 12-0459-42), anti-human CD33 (eBioscience, Thermo Fisher Scientific, catalog no. 61-0338-42) and anti-human LILRB1 (HP-F1 clone, eBioscience, Thermo Fisher Scientific, catalog no. 62-5129-42). The samples were analysed using BD LSRFortessa™ Cell Analyzer (BD Biosciences).

**WESTERN BLOTTING**

Whole cell extracts from malignant cell lines were prepared in Triton X-100-based buffer and subjected to western blotting as previously described in^2^. For LILRB1 protein detection anti human-LILRB1 antibody was used (LILRB1/CD85j; D4L8L Rabbit monoclonal Ab, Cell Signaling Technology, cat. no. 78144).

**STATISTICAL ANALYSIS AND DATA VISUALIZATION**

In figure legends, n numbers determine the number of technical replicates (MS and CFU analysis) or individual samples (patients, healthy donors of blood/PBMC, animals). All statistical analyses were conducted using GraphPad Prism 7 Software (La Jolla, CA, USA). Calculated *P* values were considered statistically significant when below 0.05 with the type of test used for statistical assessment provided in Figure legends. Two-sided tests were applied with appropriate corrections for multiple comparisons as stated in the Figure's description. The exclusion criteria were pre-established; thus, samples or experiments were only excluded from the analysis when a positive or negative control did not provide the expected results. Flow cytometry data was visualized using FlowJo™ Software (BD Life Sciences).

**REFERENCES**

1 Fidyt K, Pastorczak A, Cyran J, Crump NT, Goral A, Madzio J *et al.* Potent, p53-independent induction of NOXA sensitizes MLL-rearranged B-cell acute lymphoblastic leukemia cells to venetoclax. *Oncogene* 2022; **41**: 1600–1609.

2 Fidyt K, Pastorczak A, Goral A, Szczygiel K, Fendler W, Muchowicz A *et al.* Targeting the thioredoxin system as a novel strategy against B‐cell acute lymphoblastic leukemia. *Mol Oncol* 2019; **13**: 1180–1195.

3 Kalina T, Flores-Montero J, van der Velden VHJ, Martin-Ayuso M, Böttcher S, Ritgen M *et al.* EuroFlow standardization of flow cytometer instrument settings and immunophenotyping protocols. *Leukemia* 2012; **26**: 1986–2010.

4 Wälchli S, Løset GÅ, Kumari S, Nergård Johansen J, Yang W, Sandlie I *et al.* A Practical Approach to T-Cell Receptor Cloning and Expression. *PLoS ONE* 2011; **6**: e27930.
